# Structure–Activity Relationships of 1,4‐Benzodiazepines Inhibiting the Voltage‐Gated Potassium Channel K_V_7.1

**DOI:** 10.1002/ardp.70370

**Published:** 2026-10-23

**Authors:** Florian Roßner, Amy Methaq, Guiscard Seebohm, Bernhard Wünsch

**Affiliations:** ^1^ GRK 2515, Chemical biology of ion channels (Chembion) Universität Münster Münster Germany; ^2^ Institute of Pharmaceutical and Medicinal Chemistry University of Münster Münster Germany; ^3^ Department of Cardiovascular Medicine, Institute for Genetics of Heart Diseases (IfGH) University Hospital Münster Münster Germany

**Keywords:** 3‐amino‐1,4‐benzodiazepine‐2‐ones, chiral HPLC, K_V_7.1 inhibition, structure–activity relationships, two‐electrode voltage‐clamp, Voltage gated K^+^ channel K_V_7.1

## Abstract

K_V_7.1 antagonists hold potential for the treatment of diarrhea, reflux esophagitis, and hypoinsulinemia. The 1,4‐benzodiazepine L768,673 ((*R*)‐**1**) is a potent and selective K_V_7.1 channel blocker with a higher apparent affinity for the K_V_7.1 ion channel than other K_V_7.1 modulators including (R)‐L3. In this study, L768,673 ((*R*)‐**1**) was systematically modified at two positions, and the inhibitory effects of the resulting 1,4‐benzodiazepines were evaluated by two‐electrode voltage‐clamp (TEVC) experiments. Acylation of the primary amines *rac*‐**7** and **9** provided amides **8** and **10a**–**e**. Alkylation of secondary lactam **10a** led to tertiary lactams **12a**–**c**. Secondary lactams **10a**–**e** and tertiary lactams **12a**,**b** with large 1‐substituents could not inhibit the K_V_7.1 channel. However, racemic 3‐fluoropropyl derivative **12c** was as active as the enantiomerically pure lead compound (*R*)‐**1** rendering **12c** an interesting starting point for the development of an 18‐fluorine labeled PET tracer.

## Graphical abstract

K_V_7.1 antagonists hold potential for the treatment of diarrhea, reflux esophagitis, and hypoinsulinemia. To derive structure–activity relationships, the lead K_V_7.1 antagonist L768,673 was thoroughly modified in 1‐ and 3‐positions. The 3‐fluoropropyl‐substituted 1,4‐benzodiazepine showed promising K_V_7.1 inhibitory activity, qualifying it as a potential 18‐fluorine‐labeled PET tracer to image K_V_7.1 ion channels.

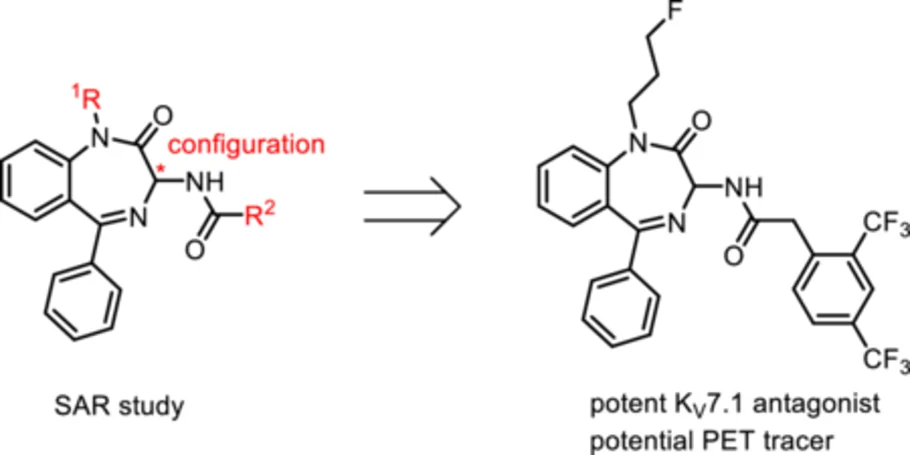

**Abbreviations**
EC_50_
: half‐maximal effective concentration
HPLC: high‐performance liquid chromatography
IC_50_
: half‐maximal inhibitory concentration
JLNS: Jervell and Lange Nielsen syndrome
LQT1: long QT‐syndrome 1
PD: pore domain
PET: positron emission tomography
S: segment
SAR: structure‐activity‐relationship
TEVC: two‐electrode‐voltage‐clamp
VSD: voltage sensing domain

## 1 Introduction

The voltage‐gated potassium channel K_V_7.1 is expressed ubiquitously in the human body [1]. It assembles as a homotetramer of 4 KCNQ1‐α‐subunits. Each α‐subunit consists of two domains, the pore domain (PD) and the voltage‐sensing‐domain (VSD). Segments S1–S4 form the VSD and S5 and S6 form the PD [2, 3]. Loss‐of‐function mutations of the K_V_7.1 ion channel are well known causes of inherited diseases such as long QT1‐(LQT1) syndrome [4] and Jervell and Lange Nielsen syndrome (JLNS) [5]. Patients with LQT1‐syndrome have a prolonged QT interval, which predisposes them to malignant arrhythmia and sudden cardiac death [6]. JLNS patients not only have the same prolonged QT interval but also have hearing loss [5]. These conditions might improve upon treatment with a K_V_7.1 agonist such as (R)‐L3 [7, 8] (Figure 1). Very recently, we reported a detailed structure–activity relationship (SAR) study on the 1,4‐benzodiazepine‐based potent agonist (R)‐L3 [9] and an in‐depth analysis of the effects exerted by some (*R*)‐configured (R)‐L3 analogs on the channel kinetics [10].

**Figure 1:**
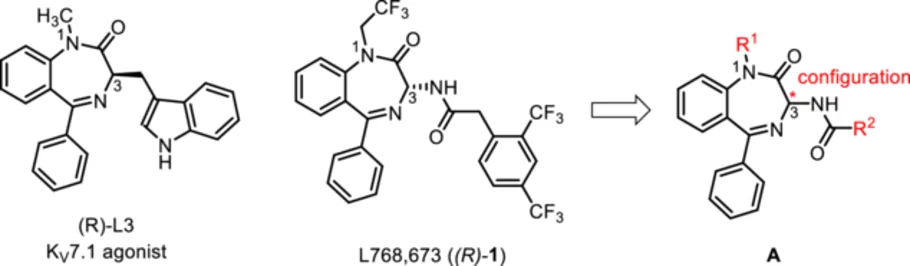
K_V_7.1 agonist (R)‐L3 and K_V_7.1 antagonist L768,673 ((*R*)‐**1**) as lead compounds for the development of novel K_V_7.1 antagonists. Envisaged structural modifications are summarized in general formula **A**. Although the orientations of the substituents in 3‐postion of the agonist (R)‐L3 and the antagonist L768,673 ((*R*)‐**1**) are different, both compounds are (3 *R*)‐configured according to the CIP formalism.

**Figure 2:**
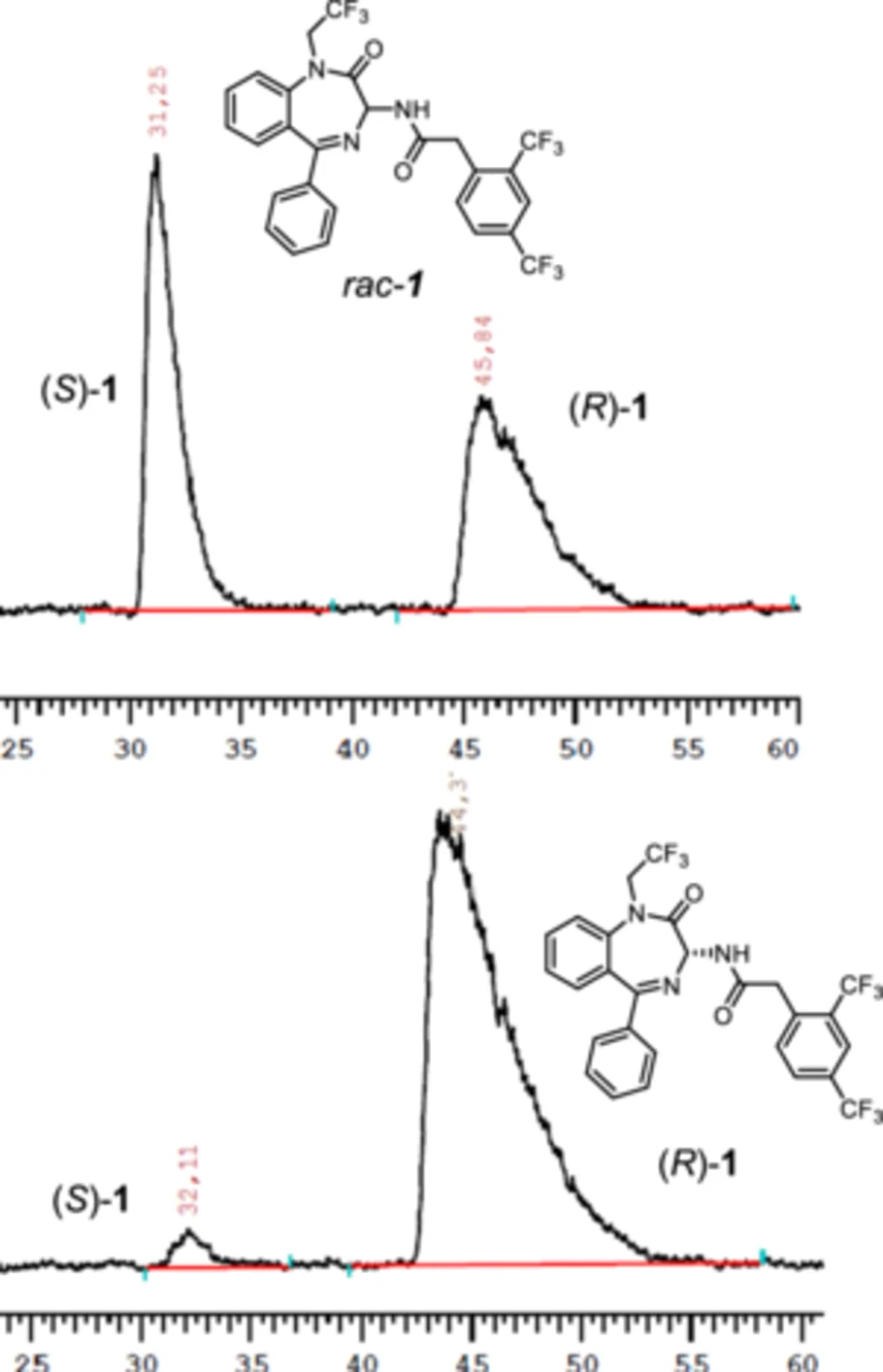
Chiral HPLC chromatograms of racemic mixture *rac*‐**1** (top) and enantiomer (*R*)‐**1** (bottom). HPLC‐conditions: Daicel Chiralpak IM (*l* = 250 mm, *d* = 4.6 mm); eluent: *iso*‐hexane/*iso*‐propanol = 97:3; flow rate: 1.0 mL/min; detection *λ *= 254 nm.

On the other hand, an inhibition of the K_V_7.1 K^+^ channel might also be potentially suited to treat certain human conditions. Cl^–^ secretion into the lumen from colon epithelial cells results in an osmotic gradient, which is the main trigger of fluid and thus feces secretion [11]. However, Cl^–^ secretion will lead to a depolarization of the colon epithelial cells and limit the electrical driving force for further Cl^–^ secretion. This effect is compensated by the K_V_7.1/KCNE3 complex in colon epithelial cells. This ion channel allows a constant efflux of K^+^ ions, which counteracts depolarization, stabilizes the membrane potential, and thus keeps the electrical driving force for further Cl^–^ secretion into the lumen [12, 13]. Therefore, an inhibition of the Kv7.1 ion channel could limit fluid and feces secretion by preventing further Cl^–^‐efflux [14]. In fact, the K_V_7.1 inhibitor chromanol 293B has been shown to inhibit secretory currents induced by cholera toxin [15], demonstrating the antidiarrheal potential of K_V_7.1 antagonists.

K_V_7.1 antagonists could also serve as a treatment option for reflux esophagitis. The H^+^/K^+^ ATPase is responsible for HCl secretion of parietal cells and pumps H^+^ into the lumen, while taking up K^+^ ions into the parietal cell. This mechanism requires a recycling of K^+^ ions, that is, a K^+^ efflux from the parietal cell to the gastric lumen, which occurs primarily through K_V_7.1 channels. Without recycling of K^+^ ions from the parietal cells into the gastric lumen, the H^+^/K^+^ exchange would limit itself due to depolarization and increased K^+^ concentration in parietal cells. Since the H^+^/K^+^ ATPase is dependent on the K_V_7.1 channel for proper function, inhibitors of the K_V_7.1 channel lead to reduced secretion of HCl into the gastric lumen [16].

K_V_7.1 overexpression has been shown to limit insulin secretion in the mouse pancreatic beta cell line MIN6 [17]. The reduced insulin secretion might be explained by increased hyperpolarization due to increased K^+^ efflux, which reduces the activity of voltage‐dependent Ca^2+^ channels. However, Ca^2+^ influx through voltage‐dependent Ca^2+^ channels is crucial for insulin secretion. Thus, reduced Ca^2+^ influx leads to an attenuated insulin secretion [18]. Therefore, K_V_7.1 antagonists could be useful tool compounds for studying insulin secretion. Moreover, they represent a potential option to increase insulin secretion [19].

Herein we report a SAR study on K_V_7.1 antagonists starting from the lead K_V_7.1 antagonist L768,673 ((*R*)‐**1**). As demonstrated by the general formula **A**, 3‐substituted 1,4‐benzodiazepin‐2‐ones with different substituents R^1^ and R^2^ were envisaged. Moreover, the configuration in 3‐position should be considered (Figure 1). The K_V_7.1 channel inhibitory activity of the resulting ligands should be tested in two‐electrode‐voltage‐clamp (TEVC) experiments.

K_V_7.1 inhibitors with a 1,4‐benzodiazepine‐scaffold are described to affect the K_V_7.1 channel at much lower concentrations than the K_V_7.1 agonist (R)‐L3. Whereas (R)‐L3 has an EC_50_‐value of 4.2 µM [20], the 1,4‐benzodiazepine L‐735821 has an IC_50_‐value of 173 nM [21]. Since activity at the K_V_7.1 channel at lower concentrations is associated with higher affinity, antagonists such as L768,673 ((*R*)‐**1**) are well suited as lead compounds for the development of a K_V_7.1 PET tracer.

## 2 Results and Discussion

### 2.1 Synthesis

Racemic 3‐amino‐1,4‐benzodiazepine *rac*‐**7** was synthesized as the key building block for the synthesis of the lead compound L768,673 ((*R*)‐**1**) and the phenol analog **8**. For the synthesis of 3‐amino‐1,4‐benzodiazepine *rac*‐**7**, 2‐aminobenzophenone (**2**) was reacted with 2‐bromoacetyl bromide to obtain the 2‐bromoacetamide **3** [22]. The bromide of **3** was substituted with methenamine in a Delépine reaction [23]. After hydrolysis of the quaternary ammonium intermediate, the resulting primary amine reacted intramolecularly with the ketone forming the 1,4‐benzodiazepinone **4** [24]. The secondary lactam **4** was deprotonated in 1‐position and then reacted with 2,2,2‐trifluoroethyl trifluoromethanesulfonate to obtain the tertiary lactam **5**. Deprotonation in α‐position to the carbonyl moiety with LiHMDS and subsequent reaction with 2,4,6‐triisopropylbenzenesulfonyl azide resulted in the formation of the azide **6** [25]. Reduction of the azide **6** with H_2_ and Pd/C led to the primary amine *rac*‐**7** [26] (Scheme 1).

Acylation of the primary amine *rac*‐**7** with acid chlorides generated in situ from the corresponding acids with (COCl)_2_ [27] led to the phenylacetamides *rac*‐**1** and **8**. (*R*)‐Configured 3‐amino‐1,4‐benzodiazepine (*R*)‐**7** was obtained by reaction of the racemic primary amine *rac*‐**7** with (*R*)‐mandelic acid and 3,5‐dichloro‐2‐hydroxybenzaldehyde. (*R*)‐Mandelic acid formed a salt with (*R*)‐configured primary amine (*R*)‐**7**, which was poorly soluble in isopropyl acetate and precipitated. (*S*)‐Configured primary amine (*S*)‐**7** reacted with 3,5‐dichloro‐2‐hydroxybenzaldehyde to form an imine, which was prone to racemization. Released (*R*)‐**7** from the imine reacted again with (*R*)‐mandelic acid to form the insoluble (*R*,*R*)‐configured salt shifting the equilibrium to (*R*)‐**7**. After separation of the (*R*,*R*)‐configured salt and removal of the auxiliary (*R*)‐mandelic acid, (*R*)‐**7** was isolated in 42% yield [27]. The (*R*)‐configured 3‐amino‐1,4‐benzodiazepine (*R*)‐**7** was then transformed into the (*R*)‐configured phenylacetamide (*R*)‐**1** (L768,673) [27] (Scheme 1).

Commercially available racemic 3‐amino‐1,4‐benzodiazepine **9** was used as a building block for the synthesis K_V_7.1 antagonists with different acyl moieties at the amine in the 3‐position. For the synthesis of amides **10a**–**d,** the primary amine **9** was acylated with various acids using the coupling reagent HBTU [28]. The 3‐nitrobenzamide **10e** was obtained by acylation of **9** with the corresponding acid chloride (Scheme 2).

**Scheme 1:**
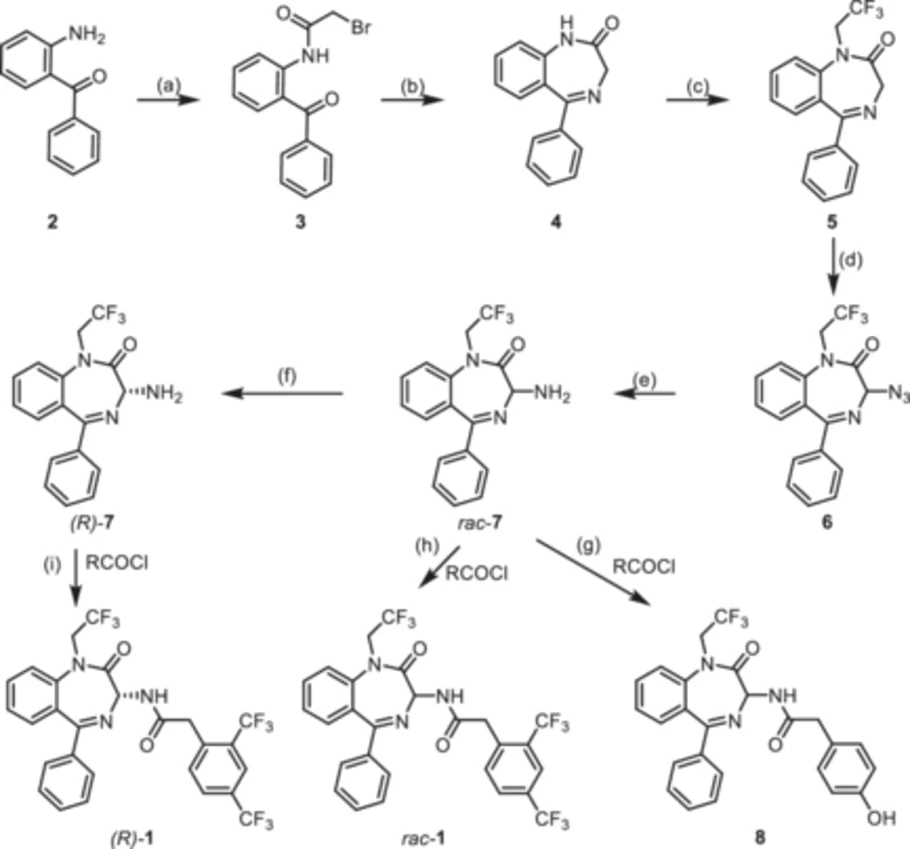
Synthesis of phenylacetamides *rac*‐**1**, (*R*)‐**1** and **8**. Reagents and reaction conditions: (a) 2‐bromoacetyl bromide, K_2_CO_3_, CH_3_CN, 0°C then rt, 30 min, 97%. (b) 1. methenamine, ammonium acetate, EtOH, reflux, 2. H_2_O, 60°C, 40 min, 88%. (c) 1. NaH, DMSO, rt 30 min, 2. 2,2,2‐trifluoroethyl trifluoromethanesulfonate (CF_3_CH_2_OSO_2_CF_3_), rt, 30 min, 78%. (d) 1. LiHMDS, THF, –80°C, 15 min, 2. 2,4,6‐triisopropylbenzensulfonyl azide, THF, –80°C, 20 min, 3. AcOH, 30°C, 2 h, 68%. (e) H_2_ (1 bar), Pd/C, EtOH, THF, rt, 2 h, 97%. (f) 3,5‐dichloro‐2‐hydroxybenzaldehyde, (*R*)‐mandelic acid, isopropyl acetate, H_2_O, rt, overnight, 42%, (*R*)‐**7**. (g) 1. 2‐(4‐hydroxyphenyl)acetic acid, (COCl)_2_, isopropyl acetate, DMF (catalytic), 2. *rac**‐*** **7**, NaHCO_3_, H_2_O, rt, 1 h, 34%. (h) 2‐[2,4‐bis(trifluoromethyl)phenyl]acetic acid, (COCl)_2_, isopropyl acetate, DMF (catalytic), 2. *rac**‐*** **7**, NaHCO_3_, H_2_O, rt, 1 h, 82%. (i) 2‐[2,4‐bis(trifluoromethyl)phenyl]acetic acid, (COCl)_2_, isopropyl acetate, DMF (catalytic), 2. (*R*)*‐* **7**, NaHCO_3_, H_2_O, rt, 1 h, 77%.

**Scheme 2:**
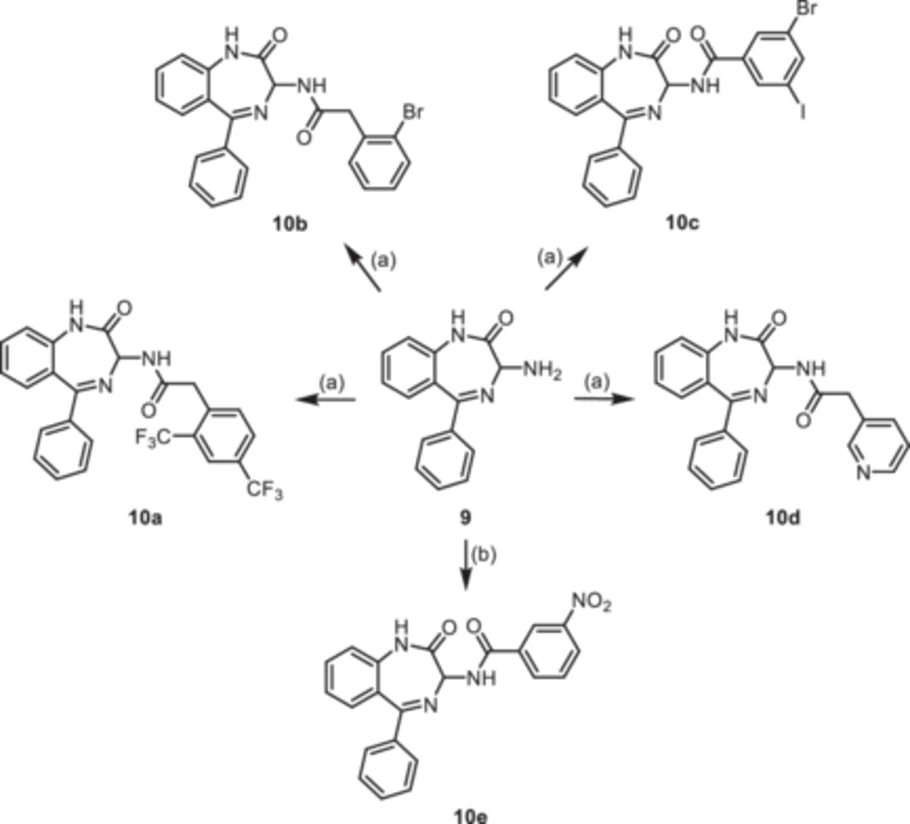
Synthesis of amides **10a**–**e**. Reagents and reaction conditions: (a) RCO_2_H, Et_3_N, HBTU, CH_2_Cl_2_/DMF 9:2, rt, overnight, 13%–78%. (b) 3‐nitrobenzoyl chloride, Et_3_N, THF/DMF 2:1, 0°C then rt, overnight, 68%.

To broaden the SAR study, various alkyl substituents were introduced at the lactam N‐atom. For this purpose, lactam **10a** was deprotonated with NaH [29] and subsequently treated with alkyl tosylates **11a**–**c** providing the tertiary lactams **12a**–**c** in 31%, 33%, and 78% yield, respectively (Scheme 3). Tosylates **11a** and **11b** were obtained by reacting heptan‐1‐ol [30] and 2‐cyclohexylethan‐1‐ol [31] with tosyl chloride (see Supporting Information). (3‐Fluoropropyl) tosylate (**11c**) was obtained by a commercial provider.

**Scheme 3:**
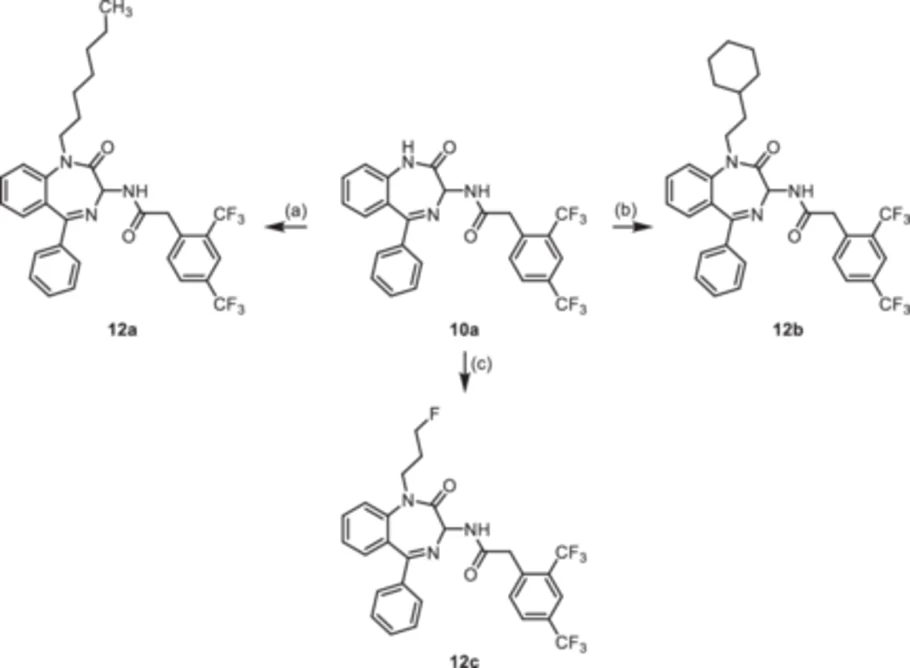
Synthesis of 1‐substituted lactams **12a**–**c**. Reagents and reaction conditions: (a) 1. NaH, THF, DMF, 0°C, 1 h; 2. heptyl tosylate (**11a**), 50°C, 3 h, 31%. (b) 1. NaH, DMF, 0°C, 1 h; 2. (2‐cyclohexylethyl) tosylate (**11b**), 40°C, 2 h, 33%. (c) 1. NaH, DMF, 0°C, 1 h; 2. (3‐fluoropropyl) tosylate (**11c**), 40°C, 1.5 h, 78%.

### 2.2 Determination of the Enantiomeric Purity of Synthesized Lead Compound (*R*)*‐*1 (L768,673)

To investigate the enantiomeric purity of isolated primary amine (*R*)‐**7** and the subsequent amide (*R*)*‐* **1** (L768,673), chiral HPLC was performed. For this purpose, the racemic mixture *rac*‐**1** was analyzed first on a Daicel Chiralpak IM column (Figure 2, top). Subsequently, the (*R*)‐enantiomer (*R*)‐**1** was analyzed using the same HPLC conditions (Figure 2, bottom). According to this analysis, the ratio of enantiomers was 96.7% (*R*)‐**1**/3.3% (*S*)‐**1**.

### 2.3 Activity of 1,4‐benzodiazepines at the K_V_7.1 Channel Measured by TEVC

Oocyte preparation and two‐electrode‐voltage‐clamp (TEVC) experiments were performed as described by Schreiber et al. [20]. In brief, cRNA encoding KCNQ1 was injected into oocytes, and the oocytes were incubated for 2–3 days to overexpress the K_V_7.1 channel. Whole‐cell currents of these oocytes were recorded at 21°C–22°C by TEVC. The effect of each compound on K_V_7.1 channels is given as the change in channel activity (in %) compared with the channel activity of the same oocyte in the presence of only ND96 buffer. The activity was determined from peak tail currents measured at −120 mV after a pulse to +40 mV. The recorded data are summarized in Table 1.

**Table 1:** Activity of synthesized 1,4‐benzodiazepines at the K_V_7.1 channel. Data in % refers to the change of channel activity in the presence of test compound (500 nM) compared to the activity in the presence of buffer only. Peak tail currents are measured at −120 mV after a pulse to +40 mV.

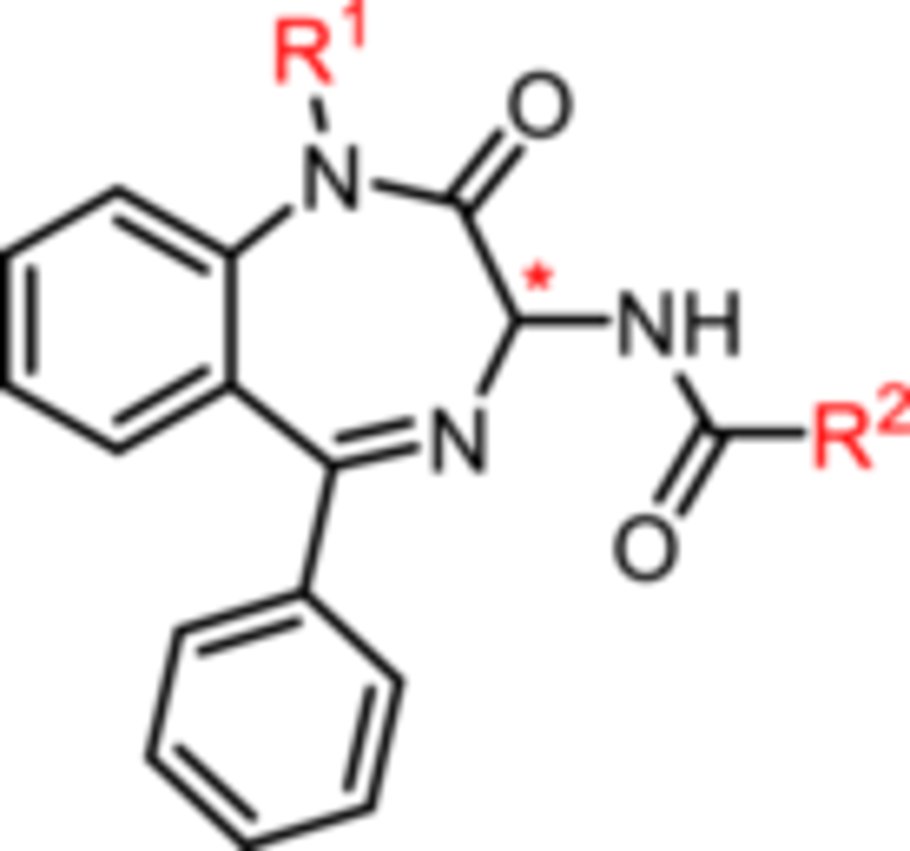				
Compd.	R^1^	R^2^	Configuration	Activity change ± SEM [%]^a^
***rac*** **‐**1	CH_2_CF_3_	2,4‐bis(trifluoromethyl)benzyl	*rac*.	−48 ± 6^b^
**(** ***R*** **)‐**1	CH_2_CF_3_	2,4‐bis(trifluoromethyl)benzyl	(*R*)	−51 ± 10
**8**	CH_2_CF_3_	4‐hydroxybenzyl	*rac*.	−30 ± 3^b^
**10a**	H	2,4‐bis(trifluoromethyl)benzyl	*rac*.	+12 ± 2
**10b**	H	2‐bromobenzyl	*rac*.	−6 ± 4
**10c**	H	3‐bromo‐5‐iodophenyl	*rac*.	−14 ± 2
**10d**	H	(pyridin‐3‐yl)methyl	*rac*.	+1 ± 1
**10e**	H	3‐nitrophenyl	*rac*.	+4 ± 7
**12a**	heptyl	2,4‐bis(trifluoromethyl)benzyl	*rac*.	−4 ± 8
**12b**	2‐cyclohexylethyl	2,4‐bis(trifluoromethyl)benzyl	*rac*.	+1 ± 4
**12c**	CH_2_CH_2_CH_2_F	2,4‐bis(trifluoromethyl)benzyl	*rac*.	−51 ± 5

^a^ Activity change of the K_V_7.1 channel in the presence of the test compound, referring to buffer only. Activity was recorded by TEVC. Values represent the mean of at least four independent experiments (*n* ≥ 4).

^b^ Compound concentration 10 µM.

The tertiary lactam (*R*)‐**1** (lead compound L768,673) strongly inhibited the K_V_7.1 channel (−51%) at a concentration of 500 nM. Removal of the trifluoroethyl moiety in 1‐position led to the (racemic) secondary lactam **10a**, which could no longer inhibit the K_V_7.1 channel. As further secondary lactams **10b**–**e** could also not antagonize the K_V_7.1 channel, it was concluded that a substituent in the 1‐position was essential to block the K_V_7.1 channel.

Therefore, starting with the secondary lactam **10a** bearing the 2,4‐bis(trifluoromethyl)benzyl moiety of the lead compound (*R*)‐**1**, various substituents were introduced at the 1‐position. Large substituents such as heptyl and 2‐cyclohexylethyl groups in the 1‐position did not lead to antagonistic effects of the 1,4‐benzodiazepines (**12a** (−4%) and **12b** (+1%). However, the smaller 3‐fluoropropyl‐substituent was well tolerated, and **12c** reduced the activity of the K_V_7.1 channel by 51%. Although **12c** represents a racemic mixture, it showed the same inhibitory activity as the enantiomerically pure lead compound (*R*)‐**1** (−51%).

## 3 Conclusion

In this study, the known K_V_7.1 inhibitor L768,673 ((*R*)‐**1**) and nine derivatives were synthesized, and their effects on K_V_7.1 channels were evaluated in TEVC experiments. It was shown that secondary lactams **10a**–**e** without a substituent at the 1‐position did not inhibit the K_V_7.1 channel, indicating a substituent in 1‐position being essential for K_V_7.1 inhibition. Large substituents in the 1‐position (**12a** (heptyl), **12b** (2‐cyclohexylmethyl)) were not tolerated by the K_V_7.1 channel. However, the smaller 3‐fluoropropyl moiety was well tolerated. The racemic mixture **12c** (–51% at 500 nM) was as active as the enantiomerically pure lead compound (*R*)‐**1** (–51% at 500 nM) and even more potent than the racemate *rac*‐**1**. Due to its primary fluoride, **12c** represents a promising starting point for the development of a fluorine‐18 labeled PET tracer suitable for imaging of K_v_7.1 channels.

## 4 Experimental

### 4.1 Chemistry

#### 4.1.1 General Methods

NMR spectroscopy: NMR spectra were recorded in deuterated solvents on Agilent DD2 400 MHz and 600 MHz spectrometers (Agilent, Santa Clara, CA, USA); chemical shifts (*δ*) are reported in parts per million (ppm) against the reference substance tetramethylsilane and calculated using the solvent residual peak of the non‐deuterated solvent; coupling constants are given with 0.5 Hz resolution; assignment of ^1^H and ^13^C NMR signals was supported by 2‐D NMR techniques where necessary. Optical rotation: Polarimeter 341 (Perkin Elmer); 1.0 dm tube; concentration *c* in g/100 mL; T = 20°C; wavelength 589 nm (d‐line of Na light); the unit of the specific rotation ([α]_D_^T^ grad. mL. dm^−1.^g^−1^) is omitted for clarity.

#### 4.1.2 HPLC Method 1 for the Determination of the Purity

Equipment 1: Pump: L‐7100, degasser: L‐7614, autosampler: L‐7200, UV detector: L‐7400, interface: D‐7000, data transfer: d‐line, data acquisition: HSM‐Software (all from Merck Hitachi, Darmstadt, Germany); Equipment 2: Pump: LPG‐3400SD, degasser: DG‐1210, autosampler: ACC‐3000T, UV‐detector: VWD‐3400RS, interface: DIONEX UltiMate 3000, data acquisition: Chromeleon 7 (equipment and software from Thermo Fisher Scientific, Lauenstadt, Germany); column: LiChrospher 60 RP‐select B (5 µm), LiChroCART 250‐4 mm cartridge; flow rate: 1.0 mL/min; injection volume: 5.0 µL; detection at *λ *= 210 nm; solvents: A: demineralized water with 0.05% (V/V) trifluoroacetic acid, B: CH_3_CN with 0.05% (V/V) trifluoroacetic acid; gradient elution (% A): 0–4 min: 90%; 4–29 min: gradient from 90% to 0%; 29–31 min: 0%; 31–31.5 min: gradient from 0% to 90%; 31.5–40 min: 90%. Unless otherwise mentioned, the purities of all test compounds are greater than 95%.

#### 4.1.3 Synthesis of *N*‐(2‐Benzoylphenyl)‐2‐Bromoacetamide (**3**)


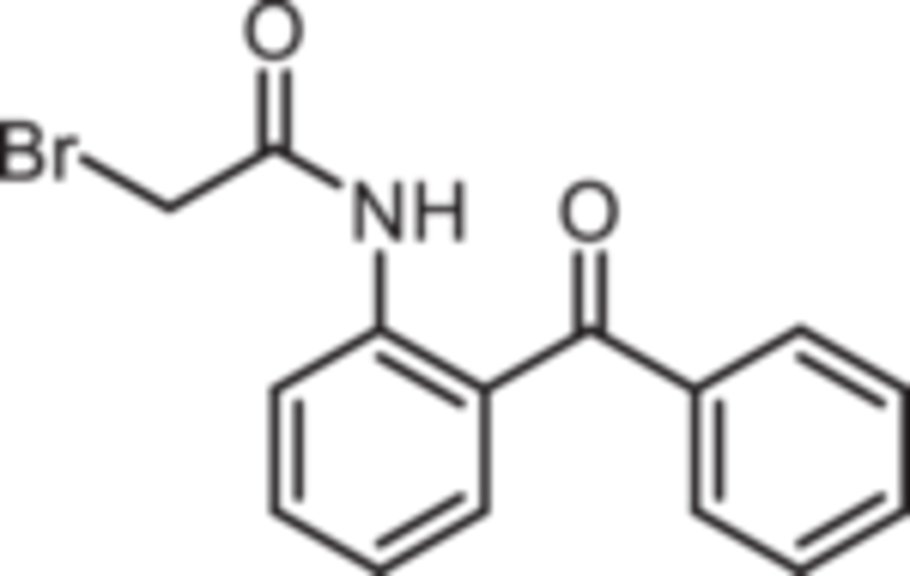


At 0°C, 2‐bromoacetyl bromide (5.50 mL, 12.78 g, 63.0 mmol, 2.0 eq.) was added over 30 min to a mixture of 2‐aminobenzophenone (**2**, 6.21 g, 31.5 mmol, 1.0 eq.) and K_2_CO_3_ (21.77 g, 157.5 mmol, 5.0 eq.) in acetonitrile (105 mL). After stirring on ice for an additional 10 min, the ice bath was removed, and the mixture was stirred for 30 min at room temperature. H_2_O (200 mL) was added, and the product was extracted with CH_2_Cl_2_ (3 × 200 mL). The organic layer was dried (Na_2_SO_4_), filtered, and concentrated in vacuo. The residue was purified by flash chromatography (SiO_2_, cyclohexane/ethyl acetate, gradient 100:0 → 0:100). Colorless solid, mp 96°C–97°C, yield 9.72 g (30.6 mmol, 97%). Purity (HPLC): 97.6% (*t*_R_ = 20.7 min). Chemical formula: C_15_H_12_BrNO_2_ (318.2 g/mol). TLC: Rf = 0.35 (cyclohexane/ethyl acetate 75:25). ^1^H NMR (600 MHz, DMSO‐d_6_): *δ* (ppm) = 4.02 (s, 2H, C*H*_2_), 7.15 (ddd, *J* = 7.9/7.3/1.1 Hz, 1H, 4‐*H*_PhNH_), 7.46–7.52 (m, 2H, 3‐*H*_Phe_, 5‐*H*_Phe_), 7.57–7.64 (m, 3H, 4‐*H*_Phe_, 3‐*H*_PhNH_, 5‐*H*_PhNH_), 7.70–7.75 (m, 2H, 2‐*H*_Phe_, 6‐*H*_Phe_), 8.60 (d, *J* = 8.2, 1H, 6‐*H*_PhNH_), 11.48 (s, 1H, N*H*). ^13^C NMR (151 MHz, DMSO‐d_6_): *δ* (ppm) = 29.6 (*C*H_2_), 121.7 (C‐6_PhNH_), 123.2 (C‐4_PhNH_), 124.2 (C‐2_PhNH_), 128.5 (2 C, C‐3_Phe_, C‐5_Phe_), 130.1 (2 C, C‐2_Phe_, C‐6_Phe_), 132.8 (C‐5_PhNH_), 133.7 (C‐3_PhNH_), 134.3 (C‐4_Phe_), 138.5 (C‐1_Phe_), 139.7 (C‐1_PhNH_), 165.1 (HN‐C═O) 199.4 ((aryl)_2_C═O). IR (neat): ν̃ (cm^−1^) = 3197 (NH, amide), 1682 (C═O), 1628 (C═C, aryl). Exact mass (APCI): *m/z* = 318.0135 (calcd 318.0124 for C_15_H_13_^79^BrNO_2_ [M + H]^+^).

#### 4.1.4 Synthesis of 5‐phenyl‐1,3‐dihydro‐1,4‐benzodiazepin‐2‐one (**4**)


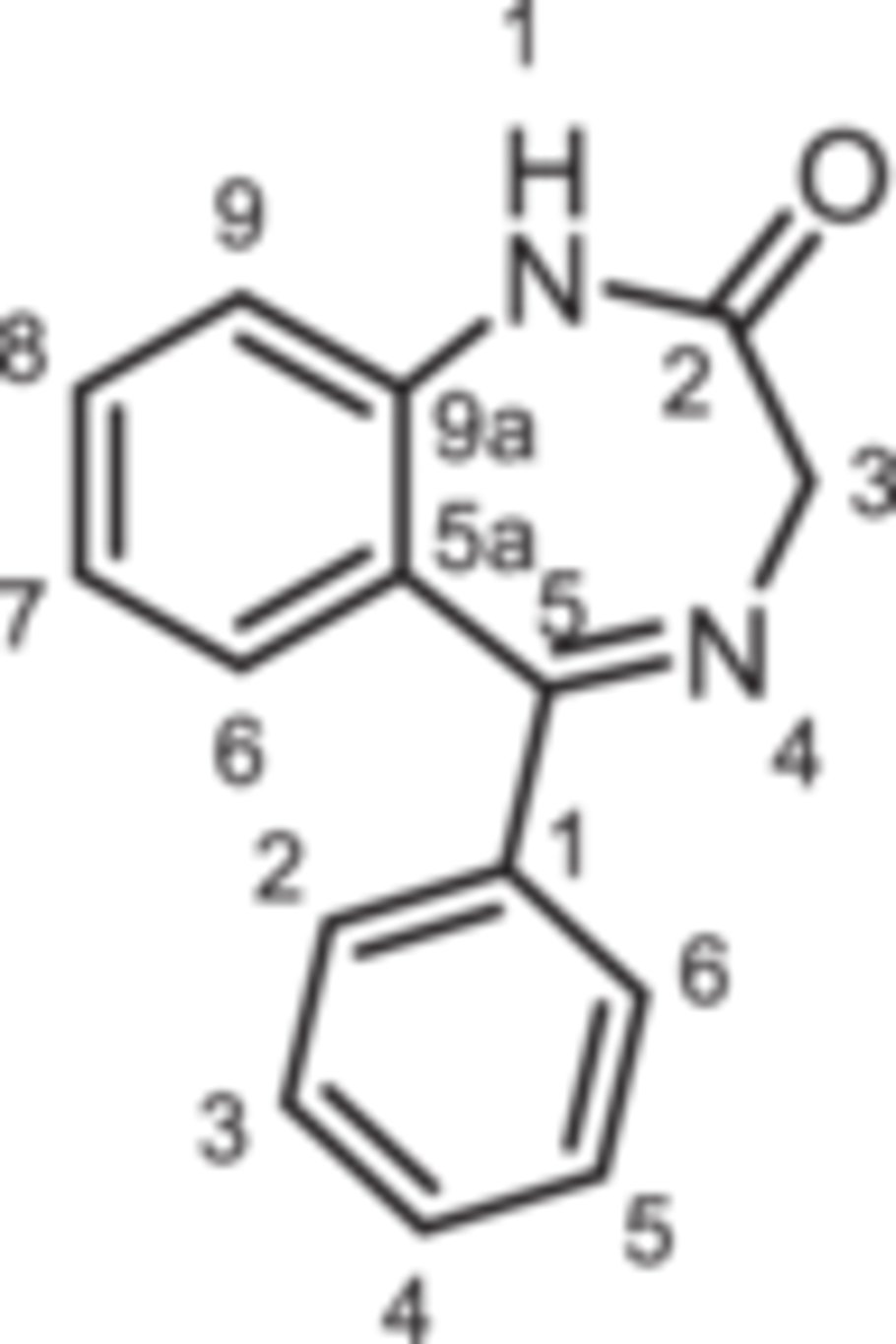


A mixture of bromoacetamide **3** (7.96 g, 25.0 mmol, 1.0 eq.), methenamine (7.01 g, 50.0 mmol, 2.0 eq.) and ammonium acetate (3.84 g, 50.0 mmol, 2.0 eq.) in EtOH (100 mL) was heated to reflux for 1.5 h. After cooling to room temperature, the mixture was concentrated in vacuo H_2_O (60 mL) was added, and the mixture was heated to 60°C for 40 min. Additional H_2_O (40 mL) was added, and the product was extracted with CH_2_Cl_2_ (3 × 100 mL). The organic layer was dried (Na_2_SO_4_), filtered, and concentrated in vacuo. The residue was purified by flash chromatography (SiO_2_, cyclohexane/ethyl acetate, gradient 100:0 → 0:100). Colorless solid, mp 184°C–185°C, yield 5.22 g (22.1 mmol, 88%). Purity (HPLC): 98.5% (*t*_R_ = 11.9 min). Chemical formula: C_15_H_12_N_2_O (236.3 g/mol). TLC: *R*_f_ = 0.21 (cyclohexane/ethyl acetate 60:40). ^1^H NMR (600 MHz, DMSO‐d_6_): *δ* (ppm) = 4.12 (s, 2H, C*H*_2bdz_), 7.17 (ddd, *J* = 8.0/7.1/1.2 Hz, 1H, 7‐*H*_bdz_), 7.23 (dd, *J* = 8.0/1.6 Hz, 1H, 6‐*H*_bdz_), 7.25 (dd, *J* = 8.2/1.2 Hz, 1H, 9‐*H*_bdz_), 7.40– 7.44 (m, 2H, 3‐*H*_Phe_, 5‐*H*_Phe_), 7.45–7.52 (m, 3H, 2‐*H*_Phe_, 4‐*H*_Phe_, 6‐*H*_Phe_), 7.56 (ddd, *J* = 8.2/7.1/1.6 Hz, 1H, 8‐*H*_bdz_), 10.54 (s, 1H, N*H*). ^13^C NMR (151 MHz, DMSO‐d_6_): *δ* (ppm) = 57.0 (*C*H_2bdz_), 121.1 (C‐9_bdz_), 122.6 (C‐7_bdz_), 126.3 (C‐5a_bdz_), 128.2 (2 C, C‐3_Phe_, C‐5_Phe_), 129.2 (2 C, C‐2_Phe_, C‐6_Phe_), 130.2 (C‐4_Phe_), 130.7 (C‐6_bdz_), 131.5 (C‐8_bdz_), 139.1 (C‐1_Phe_), 139.6 (C‐9a_bdz_), 169.7 (C‐5_bdz_), 170.4 (C═O). IR (neat): ν̃ (cm^−1^) = 3183 (N─H, amide), 3047 (C─H, aryl), 1670 (C═O), 1608 (C═C, aryl), 1188 (C─N, amide). Exact mass (APCI): *m/z* = 237.1025 (calcd. 237.1022 for C_15_H_13_N_2_O [M + H]^+^).

#### 4.1.5 Synthesis of 5‐phenyl‐1‐(2,2,2‐trifluoroethyl)‐1,3‐dihydro‐1,4‐benzodiazepin‐2‐one (**5**)


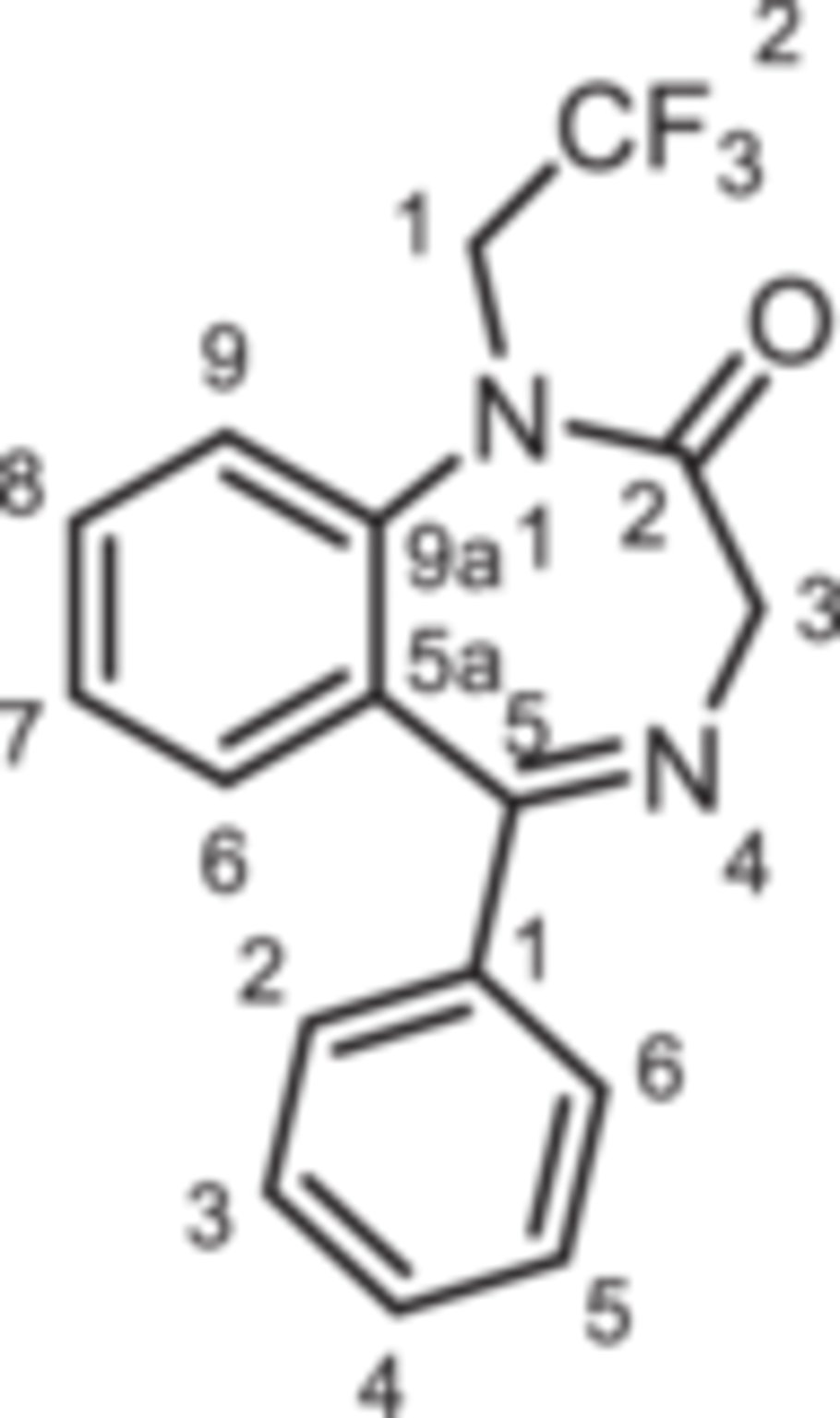


A solution of 1,4‐benzodiazepine **4** (4.26 g, 18.0 mmol, 1.00 eq.) in DMSO (80 mL) was treated with NaH (60% dispersion in paraffin, 756 mg, 18.9 mmol, 1.05 eq.) while stirring at room temperature for 30 min. 2,2,2‐Trifluoroethyl trifluoromethanesulfonate (3.90 mL, 6.27 g, 27.0 mmol, 1.50 eq.) was added, and the mixture was stirred for an additional 30 min. Then the mixture was poured into a saturated solution of NH_4_Cl (40 mL). H_2_O (160 mL) was added, and the product was extracted with CH_2_Cl_2_ (3 × 200 mL). The organic layer was dried (Na_2_SO_4_), filtered, and concentrated in vacuo. The residue was purified by flash chromatography (SiO_2_, cyclohexane/ethyl acetate, gradient 100:0 → 0:100). Colorless solid, mp 138°C–139°C, yield 4.47 g (14.0 mmol, 78%). Purity (HPLC): 98.8% (*t*_R_ = 18.5 min). Chemical formula: C_17_H_13_F_3_N_2_O (318.3 g/mol). TLC: *R*_f_ = 0.38 (cyclohexane/ethyl acetate 60:40). ^1^H NMR (600 MHz, DMSO‐d_6_): *δ* (ppm) = 3.82 (d, *J* = 10.7 Hz, 1H, C*H*_2bdz_), 4.61 (d, *J* = 10.7 Hz, 1H, C*H*_2bdz_), 4.72 (dq, *J* = 15.9/9.3 Hz, 1H, NC*H*_2_CF_3_), 5.12 (dq, *J* = 15.7/9.5 Hz, 1H, NC*H*_2_CF_3_), 7.24 (dd, *J* = 7.8/1.6 Hz, 1H, 9‐*H*_bdz_), 7.35 (td, *J* = 7.6/1.1 Hz, 1H, 8‐*H*_bdz_), 7.43–7.55 (m, 5H, 2‐*H*_Phe_, 3‐*H*_Phe_, 4‐*H*_Phe_, 5‐*H*_Phe_, 6‐*H*_Phe_), 7.68 (td, *J* = 8.5/1.6 Hz, 1H, 7‐*H*_bdz_), 7.81 (d, *J* = 8.5 Hz, 1H, 6‐*H*_bdz_). ^13^C NMR (151 MHz, DMSO‐d_6_): *δ* (ppm) = 45.4 (q, *J* = 32.8 Hz, N*C*H_2_CF_3_), 56.0 (*C*H_2bdz_), 122.8 (C‐6_bdz_), 123.4 (q, *J* = 280.4 Hz, NCH_2_ *C*F_3_), 125.4 (C‐8_bdz_), 128.4 (2 C, C‐3_Phe_, C‐5_Phe_), 128.8 (2C, C‐2_Phe_, C‐6_Phe_), 129.6 (C‐9_bdz_), 129.7 (C‐9a_bdz_), 130.5 (C‐4_Phe_), 131.7 (C‐7_bdz_), 138.3 (C‐1_Phe_), 141.2 (C‐5a_bdz_), 169.5 (C═O), 169.8 (C‐5_bdz_). IR (neat): ν̃ (cm^−1^) = 3062 (C‐H, aryl), 1678 (C═O), 1605 (C═C, aryl), 1146 (C─N, amide). Exact mass (APCI): *m/z* = 319.1056 (calcd. 319.1053 for C_17_H_14_F_3_N_2_O [M + H]^+^).

#### 4.1.6 Synthesis of 3‐azido‐5‐phenyl‐1‐(2,2,2‐trifluoroethyl)‐1,3‐dihydro‐1,4‐benzodiazepin‐2‐one (**6**)


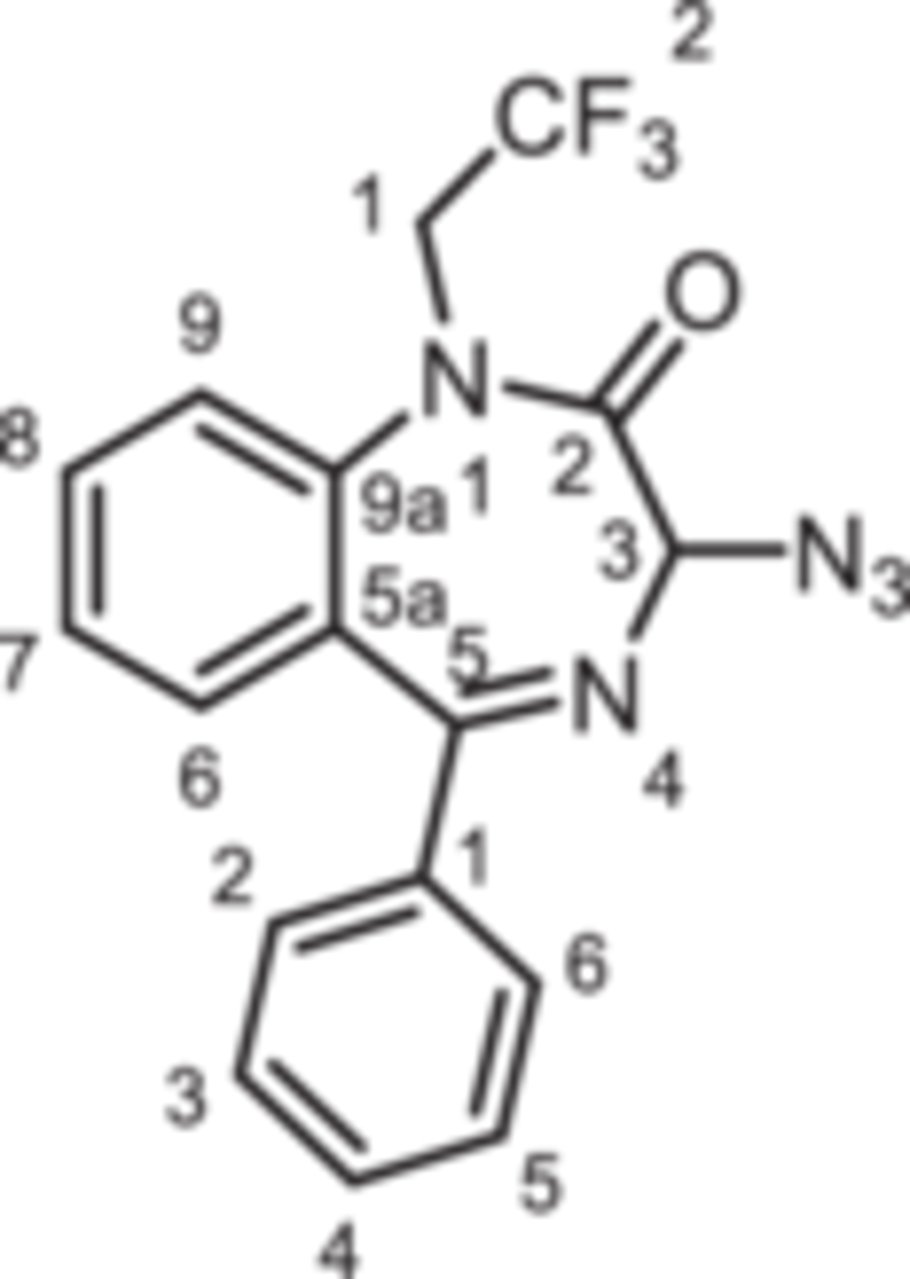


At –80°C, 1,4‐benzodiazepine **5** (3.82 g, 12.0 mmol, 1.0 eq.) dissolved in THF (70 mL) was deprotonated with LiHMDS (1 m, 12.0 mL, 12.0 mmol, 1.2 eq.). Stirring was continued for 15 min and 2,4,6‐triisopropylbenzenesulfonyl azide (7.43 g, 24.0 mmol, 2.0 eq.) in THF (20 mL) was added. The mixture was stirred for 20 min. AcOH (2.75 mL, 2.88 g, 48.0 mmol, 4.0 eq.) was added, and the mixture was stirred at 30°C for 2 h. A saturated solution of NaHCO_3_ (40 mL) was added slowly. Additional H_2_O (160 mL) was added, and the product was extracted with CH_2_Cl_2_ (3 × 200 mL). The organic layer was dried (Na_2_SO_4_), filtered, and concentrated in vacuo. The residue was purified by flash chromatography (SiO2, cyclohexane/ethyl acetate, gradient 100:0 → 0:100). Colorless solid, mp 167°C–168°C, yield 2.95 g (8.2 mmol, 68%). Purity (HPLC): 97.4% (*t*_R_ = 23.0 min). Chemical formula: C_17_H_12_F_3_N_5_O (359.3 g/mol). TLC: Rf = 0.36 (cyclohexane/ethyl acetate 70:30). ^1^H NMR (600 MHz, DMSO‐d6): *δ* (ppm) = 4.83 (dq, *J* = 15.8/9.4 Hz, 1H, NC*H*_2_CF_3_), 4.93 (s, 1H, 3‐*H*_bdz_), 5.13 (dq, *J* = 15.7/9.5 Hz, 1H, NC*H*_2_CF_3_), 7.31 (dd, *J* = 7.9/1.6 Hz, 1H, 9‐*H*_bdz_), 7.40 (td, *J* = 7.6/1.0 Hz, 1H, 8‐*H*_bdz_), 7.50–7.54 (m, 4H, 2‐*H*_Phe_, 3‐*H*_Phe_, 5‐*H*_Phe_, 6‐*H*_Phe_), 7.55–7.61 (m, 1H, 4‐*H*_Phe_), 7.74 (td, *J* = 8.6/1.6 Hz, 1H, 7‐*H*_bdz_), 7.85 (dd, J = 8.4/1.0 Hz, 1H, 6‐*H*_bdz_). ^13^C NMR (151 MHz, DMSO‐d6): *δ* (ppm) = 46.1 (q, *J* = 32.8 Hz, N*C*H_2_CF_3_), 75.2 (*C*HN_3_), 123.4 (C‐6_bdz_), 125.1 (q, *J* = 282.5 Hz, NCH_2_ *C*F_3_), 126.0 (C‐8_bdz_), 128.7 (2 C, C‐3_Phe_, C‐5_Phe_), 129.1 (2 C, C‐2_Phe_, C‐6_Phe_), 129.7 (2C, C‐9a_bdz_, C‐9_bdz_), 131.2 (C‐4_Phe_), 132.5 (C‐7_bdz_), 137.2 (C‐1_Phe_), 140.0 (C‐5a_bdz_), 167.1 (C═O), 167.5 (C‐5_bdz_). IR (neat): ν̃ (cm^−1^) = 3070 (C‐H, aryl), 2118 (N_3_), 1690 (C═O), 1153 (C‐N, amide). Exact mass (APCI): *m/z* = 360.1059 (calcd. 360.1067 for C_17_H_13_F_3_N_5_O [M + H]^+^).

#### 4.1.7 Synthesis of 3‐amino‐5‐phenyl‐1‐(2,2,2‐trifluoroethyl)‐1,3‐dihydro‐1,4‐benzodiazepin‐2‐one (*rac‐* **7**)


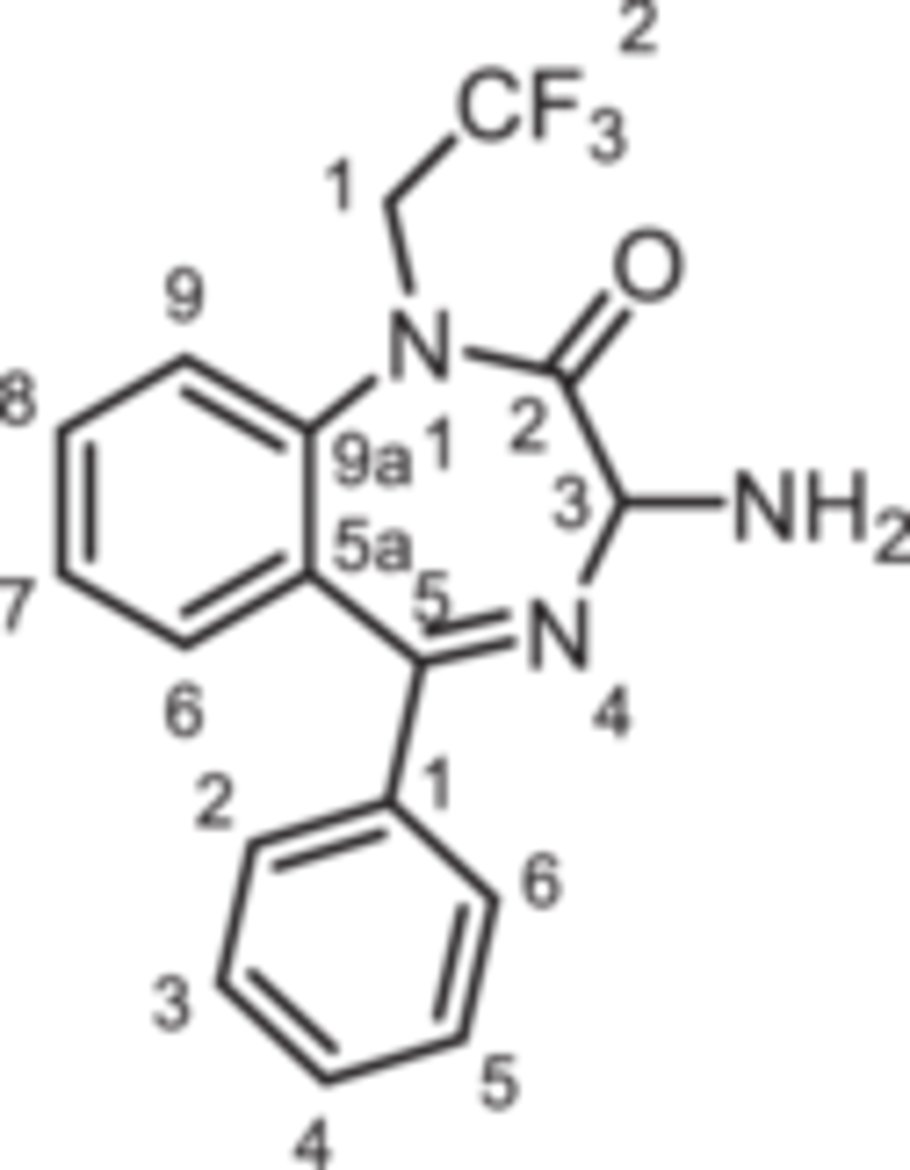


3‐Azido‐1,4‐benzodiazepine **6** (2.69 g, 7.5 mmol, 1.0 eq.) was dissolved in EtOH (85 mL) and THF (57 mL). Pd/C (20 wt%, 540 mg) was added. Air was exchanged with an H_2_ atmosphere, and a balloon filled with H_2_ was connected to the flask. The mixture was stirred at room temperature under atmospheric pressure for 9 h. The suspension was filtered, and the filtrate was concentrated in vacuo. The residue was purified by flash chromatography (SiO_2_, CH_2_Cl_2_/MeOH, gradient 100:0 → 85:15). Colorless solid, mp 142°C, yield 2.42 g (7.3 mmol, 97%). Purity (HPLC): 99.5% (*t*_R_ = 16.4 min). Chemical formula: C_17_H_14_F_3_N_3_O (333.3 g/mol). TLC: *R*_f_ = 0.26 (CH_2_Cl_2_/MeOH 100:4.5). ^1^H NMR (600 MHz, DMSO‐d_6_): *δ* (ppm) = 2.60 (s, 2H, N*H*_2_), 4.40 (s, 1H, 3‐*H*_bdz_), 4.78 (dq, *J* = 16.0/9.5 Hz, 1H, NC*H*_2_CF_3_), 5.12 (dq, *J* = 15.7/9.5 Hz, 1H, NC*H*_2_CF_3_), 7.26 (dd, *J* = 7.8/1.6 Hz, 1H, 9‐*H*_bdz_), 7.35 (td, *J* = 7.6/1.1 Hz, 1H, 8‐*H*_bdz_), 7.42–7.49 (m, 5H, 2‐*H*_Phe_, 3‐*H*_Phe_, 4‐*H*_Phe_, 5‐*H*_Phe_, 6‐*H*_Phe_), 7.69 (ddd, *J* = 8.5/7.3/1.6 Hz, 1H, 7‐*H*_bdz_), 7.84 (dd, *J* = 8.4/1.1 Hz, 1H, 6‐*H*_bdz_). ^13^C NMR (151 MHz, DMSO‐d_6_): *δ* (ppm) = 45.9 (q, *J* = 32.6 Hz, N*C*H_2_CF_3_), 70.2 (C‐3_bdz_), 122.9 (C‐6_bdz_), 125.3 (q, *J* = 279.4 Hz, NCH_2_ *C*F_3_), 125.5 (C‐8_bdz_), 128.4 (2 C, C‐3_Phe_, C‐5_Phe_), 128.8 (2C, C‐2_Phe_, C‐6_Phe_), 129.1 (C‐9_bdz_), 130.1 (C‐9a_bdz_), 130.4 (C‐4_Phe_), 131.9 (C‐7_bdz_), 137.9 (C‐1_Phe_), 140.4 (C‐5a_bdz_), 164.9 (C‐5_bdz_), 170.6 (C═O). IR (neat): ν̃ (cm^−1^) = 3414 (NH_2_), 3070 (C─H, aryl), 1690 (C═O), 1601 (C═C, aryl), 1169 (C─N, amide). Exact mass (APCI): *m/z* = 334.1146 (calcd. 334.1162 for C_17_H_15_F_3_N_3_O [M + H]^+^).

#### 4.1.8 Synthesis of (3 *R*)‐3‐amino‐5‐phenyl‐1‐(2,2,2‐trifluoroethyl)‐1,3‐dihydro‐1,4‐benzodiazepin‐2‐one ((*R*)*‐* **7**)


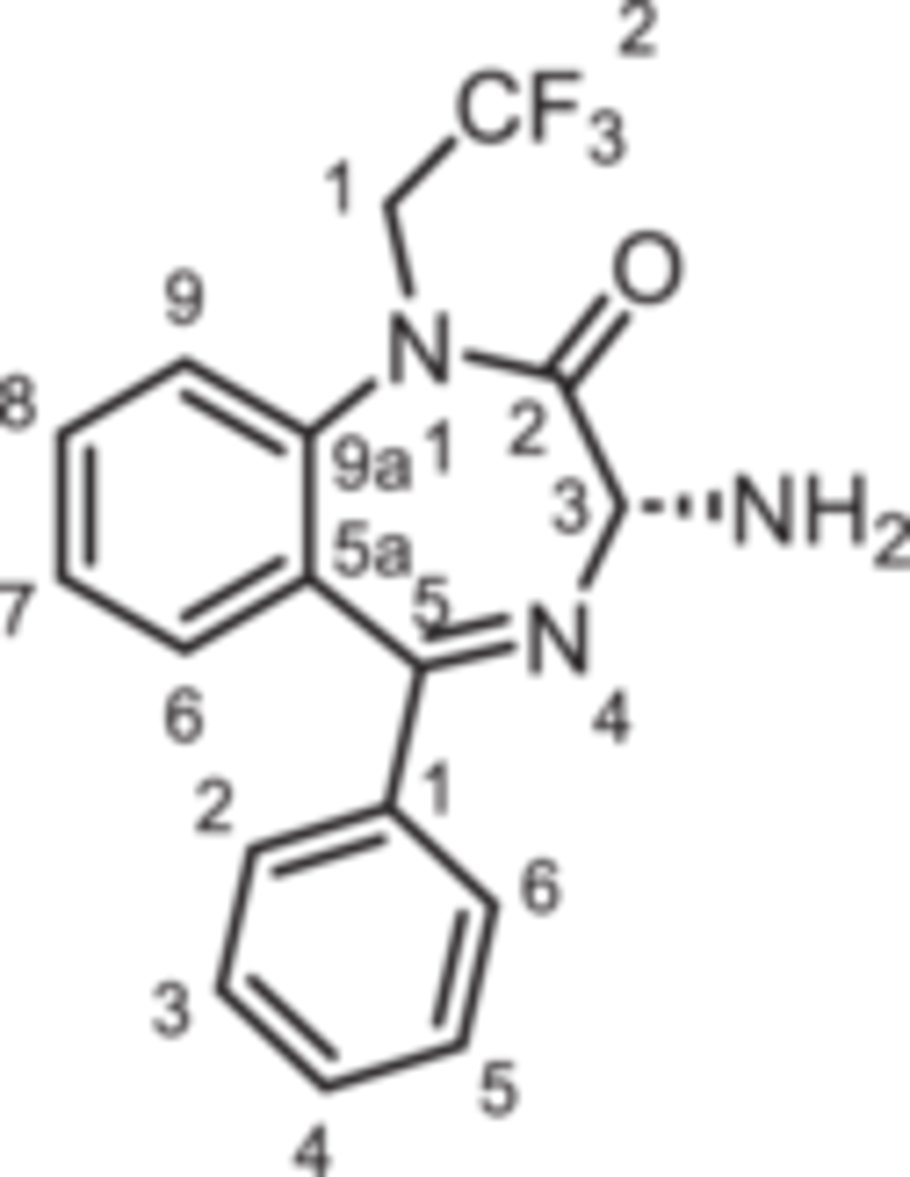


A mixture of 3‐amino‐1,4‐benzodiazepine *rac‐* **7** (1.67 g, 5.0 mmol, 1.00 eq.) and 3,5‐dichloro‐2‐hydroxybenzaldehyde (20 mg, 0.1 mmol, 0.02 eq.) in isopropyl acetate (22 mL) and H_2_O (0.2 mL) was stirred at room temperature. (*R*)‐Mandelic acid (761 mg, 5.0 mmol, 1.00 eq.) was added over 5.5 h. The mixture was stirred overnight, filtered, and the solid was washed with isopropyl acetate (5 mL). The solid was purified by flash chromatography (SiO_2_, CH_2_Cl_2_/MeOH, gradient 100:0 → 85:15). Colorless solid, mp 142°C–143°C, yield 692 mg (2.1 mmol, 42%). Purity (HPLC): 99.7% (*t*_R_ = 16.5 min). Chemical formula: C_17_H_14_F_3_N_3_O (333.3 g/mol). TLC: *R*_f_ = 0.26 (CH_2_Cl_2_/MeOH 100:4.5). ^1^H NMR, ^13^C NMR, and IR spectra are identical with the spectra of *rac‐* **7**. Exact mass (APCI): *m/z* = 334.1162 (calcd 334.1162 for C_17_H_15_F_3_N_3_O [M + H]^+^). Specific rotation: ${\left[\alpha \right]}_{20}^{D}$ = +64.4 (c = 0.24; CH_3_OH).

#### 4.1.9 Synthesis of 2‐[2,4‐bis(trifluoromethyl)phenyl]‐*N*‐[2‐oxo‐5‐phenyl‐1‐(2,2,2‐trifluoroethyl)‐2,3‐dihydro‐*1H‐1*,4‐benzodiazepin‐3‐yl]acetamide (*rac‐* **1**)


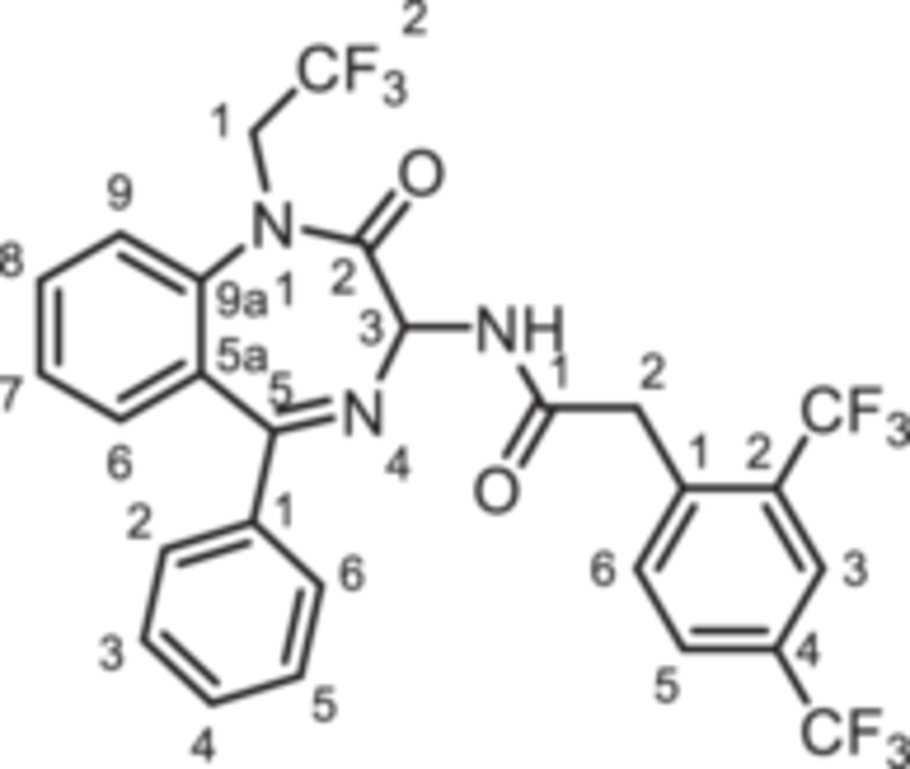


A solution of (COCl)_2_ (0.05 mL, 66 mg, 0.52 mmol,1.04 eq.) in isopropyl acetate (0.14 mL) was added over 45 min to a mixture of 2‐[2,4‐bis(trifluoromethyl)phenyl]acetic acid (0.10 mL, 142 mg, 0.52 mmol, 1.04 eq.) and DMF (two drops) in isopropyl acetate (0.14 mL). The mixture was stirred for 2 h at room temperature. The resulting acid chloride was added over 15 min to a mixture of 3‐aminobenzodiazepine *rac‐* **7** (167 mg, 0.50 mmol, 1.00 eq.) and NaHCO_3_ (224 mg, 2.67 mmol, 5.33 eq.) in H_2_O (4.00 mL) and isopropyl acetate (4.85 mL). After stirring for 1 h at room temperature, CH_2_Cl_2_ (200 mL) was added, and the organic layer was washed with saturated NaHCO_3_ (3 × 200 mL) and then H_2_O (2 × 200 mL). The organic layer was dried (Na_2_SO_4_), filtered, and concentrated in vacuo. The residue was purified by flash chromatography (SiO_2_, cyclohexane/ethyl acetate, gradient 100:0 → 0:100). Colorless solid, mp 132°C–133°C, yield 241 mg (0.41 mmol, 82%). Purity (HPLC): 100.0% (*t*_R_ = 24.9 min). Chemical formula: C_27_H_18_F_9_N_3_O_2_ (587.4 g/mol). TLC: *R*_f_ = 0.21 (cyclohexane/ethyl acetate 70:30). ^1^H NMR (600 MHz, DMSO‐d_6_): *δ* (ppm) = 4.03 (s, 2H, C*H*_2_CONH), 4.79 (dq, *J* = 15.5/9.4 Hz, 1H, CF_3_C*H*_2_), 5.15 (dq, *J* = 15.5/9.4 Hz, 1H, CF_3_C*H*_2_), 5.41 (d, *J* = 7.8 Hz, 1H, 3‐*H*_bdz_), 7.31 (dd, *J* = 7.9/1.5 Hz, 1H, 9‐*H*_bdz_), 7.40 (t, *J* = 7.6, Hz, 1H, 8‐*H*_bdz_), 7.47–7.59 (m, 5H, 2‐*H*_Phe_, 3‐*H*_Phe_, 4‐*H*_Phe_, 5‐*H*_Phe_, 6‐*H*_Phe_), 7.71–7.78 (m, 2H, 6‐*H*_bdz_, 7‐*H*_bdz_), 7.87 (d, *J* = 8.3 Hz, 1H, 6‐$H_{{\left({\text{CF}}_{3}\right)}_{2}\text{Phe}}$), 7.97 (s, 1H, 3‐$H_{{\left({\text{CF}}_{3}\right)}_{2}\text{Phe}}$), 8.03 (d, *J* = 8.3 Hz, 1H, 5‐$H_{{\left({\text{CF}}_{3}\right)}_{2}\text{Phe}}$), 9.71 (d, *J* = 7.8 Hz, 1H, N*H*). ^13^C NMR (151 MHz, DMSO‐d_6_): *δ* (ppm) = 38.3 (*C*H_2_CONH), 46.0 (q, *J *= 33.6 Hz, N*C*H_2_CF_3_), 67.7 (C‐3_bdz_), 122.4 (m, C‐$3_{{\left({\text{CF}}_{3}\right)}_{2}\text{Phe}}$), 122.6 (q, *J* = 273.9 Hz, CF_3_‐C‐$4_{{\left({\text{CF}}_{3}\right)}_{2}\text{Phe}}$) 123.2 (C‐$6_{{\left({\text{CF}}_{3}\right)}_{2}\text{Phe}}$), 124.0 (q, *J* = 281.4 Hz, CF_3_‐C‐$2_{{\left({\text{CF}}_{3}\right)}_{2}\text{Phe}}$), 126.0 (C‐8_bdz_), 127.7 (q, *J* = 32.7 Hz, C‐$4_{{\left({\text{CF}}_{3}\right)}_{2}\text{Phe}}$), 128.0 (q, *J* = 280.4 Hz, NCH_2_ *C*F_3_), 128.5 (2C, C‐3_Phe_, C‐5_Phe_), 128.7 (q, *J* = 30.3 Hz, C‐$2_{{\left({\text{CF}}_{3}\right)}_{2}\text{Phe}}$), 128.9, (3 C, C‐2_Phe_, C‐6_Phe_, C‐$5_{{\left({\text{CF}}_{3}\right)}_{2}\text{Phe}}$), 129.4 (C‐9_bdz_), 129.8 (C‐5a_bdz_), 130.9 (C‐4_Phe_), 132.4 (C‐6_bdz_), 134.5 (C‐7_bdz_), 137.4 (C‐1_Phe_), 139.2 (C‐$1_{{\left({\text{CF}}_{3}\right)}_{2}\text{Phe}}$), 140.3 (C‐9a_bdz_), 167.2 (C‐5_bdz_), 167.9 (C═O_bdz_), 168.9 (C═O_acetyl_). IR (neat): ν̃ (cm^–1^) = 3059 (C─H, aryl), 1670 (C═O, amide/lactam), 1605 (C═C, aryl), 1161 (C─N, amide), 1118 (CF_3_). Exact mass (APCI): *m/z* = 588.1335 (calcd. 588.1328 for C_27_H_19_F_9_N_3_O_2_ [M + H]^+^).

#### 4.1.10 Synthesis of (*R*)‐2‐[2,4‐bis(trifluoromethyl)phenyl]‐*N*‐[2‐oxo‐5‐phenyl‐1‐(2,2,2‐trifluoroethyl)‐2,3‐dihydro‐*1H*‐1,4‐benzodiazepin‐3‐yl]acetamide ((*R*)*‐* **1**)


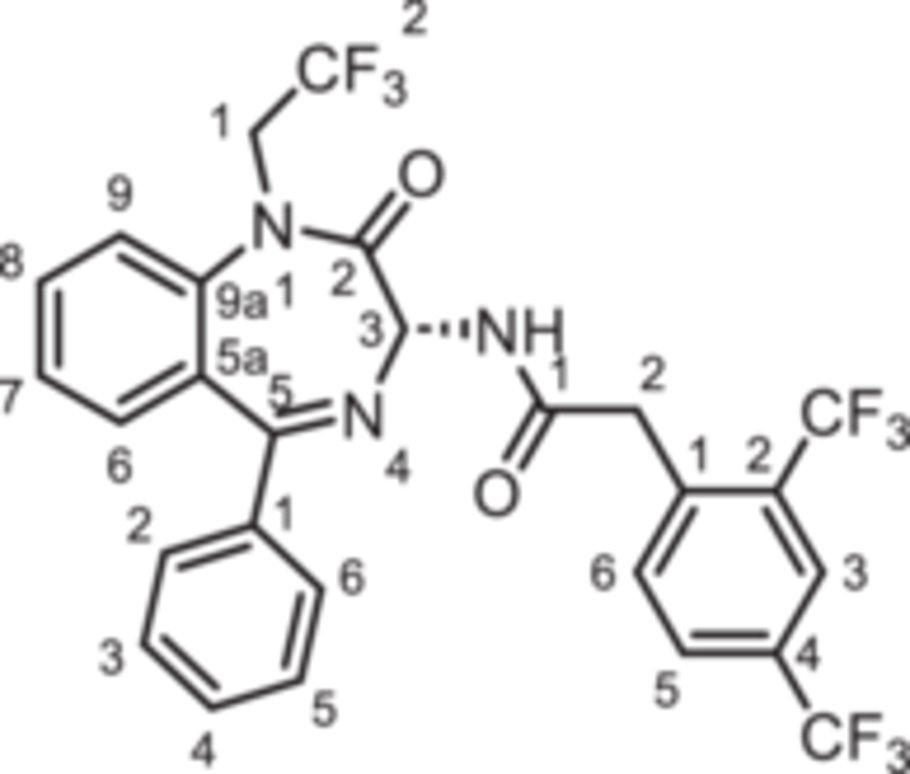


The synthesis of (*R*)‐**1** was done as described for *rac*‐**1**. ‐[2,4‐Bis(trifluoromethyl)phenyl]acetic acid (0.10 mL, 142 mg, 0.52 mmol, 1.04 eq.) was reacted with (COCl)_2_ (0.05 mL, 66 mg, 0.52 mmol,1.04 eq.) and DMF (two drops). The resulting acid chloride was reacted with (*R*)‐configured 3‐aminobenzodiazepine (*R*)‐**7** (167 mg, 0.50 mmol, 1.00 eq.). Colorless solid, mp 133°C–134°C, yield 226 mg (0.39 mmol, 77%). Purity (HPLC): 100.0% (*t*_R_ = 24.9 min). Chemical formula: C_27_H_18_F_9_N_3_O_2_ (587.4 g/mol). TLC: *R*_f_ = 0.21 (cyclohexane/ethyl acetate 70:30). ^1^H NMR, ^13^C NMR, and IR spectra are identical with the spectra of *rac‐* **11**. Exact mass (APCI): *m/z* = 588.1307 (calcd 588.1328 for C_27_H_19_F_9_N_3_O_2_ [M + H]^+^). Specific rotation: measured: ${\left[\alpha \right]}_{20}^{D}$ = −22.9 (c = 0.12; CH_3_OH), Lit.: ${\left[\alpha \right]}_{20}^{D}$ = −20.4 (c = 0.8, CH_3_OH) [27].

#### 4.1.11 Synthesis of 2‐(4‐hydroxyphenyl)‐*N*‐[2‐oxo‐5‐phenyl‐1‐(2,2,2‐trifluoroethyl)‐2,3‐dihydro‐*1H*‐1,4‐benzodiazepin‐3‐yl]acetamide (8)


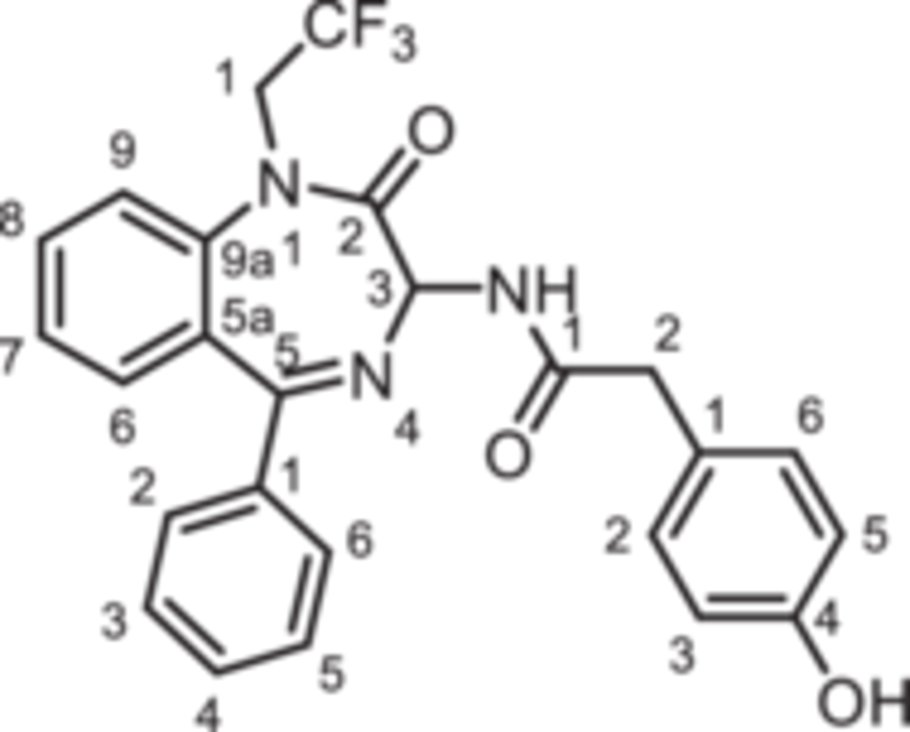


A solution of (COCl)_2_ (0.05 mL, 66 mg, 0.52 mmol,1.04 eq.) in isopropyl acetate (0.14 mL) was added over 45 min to a mixture of 2‐(4‐hydroxyphenyl)acetic acid (0.06 mL, 79 mg, 0.52 mmol, 1.04 eq.) and DMF (two drops) in isopropyl acetate (0.14 mL). The mixture was stirred for 2 h at room temperature. The resulting acid chloride was added over 15 min to a mixture of 3‐aminobenzodiazepine *rac‐* **7** (167 mg, 0.50 mmol, 1.00 eq.) and NaHCO_3_ (224 mg, 2.67 mmol, 5.33 eq.) in H_2_O (4.00 mL) and isopropyl acetate (4.85 mL). After stirring for 1 h at room temperature, ethyl acetate (200 mL) was added, and the organic layer was washed with saturated NaHCO_3_ (3 × 200 mL) and then H_2_O (2 × 200 mL). The organic layer was dried (Na_2_SO_4_), filtered, and concentrated in vacuo. The residue was purified by flash chromatography (SiO_2_, cyclohexane/ethyl acetate, gradient 100:0 → 0:100). Colorless solid, mp 140°C–141°C, yield 80 mg (0.17 mmol, 34%). Purity (HPLC): 100.0% (*t*_R_ = 19.7 min). Chemical formula: C_25_H_20_F_3_N_3_O_3_ (467.5 g/mol). TLC: Rf = 0.23 (cyclohexane/ethyl acetate 60:40). ^1^H NMR (600 MHz, DMSO‐d6): *δ* (ppm) = 3.44–3.49 (m, 2H, NHCOC*H*_2_), 4.78 (dq, *J* = 15.5/9.3 Hz, 1H, CF_3_C*H*_2_), 5.13 (dq, *J* = 15.6/9.4 Hz, 1H, CF_3_C*H*_2_), 5.38 (d, *J* = 8.1 Hz, 1H, 3‐*H*_bdz_), 6.68 (d, *J* = 8.5 Hz, 2H, 3‐*H*_Phenol_, 5‐*H*_Phenol_), 7.10 (d, *J* = 8.5 Hz, 2H, 2‐*H*_Phenol_, 6‐*H*_Phenol_), 7.31 (dd, *J* = 7.9/1.6 Hz, 1H, 9‐*H*_bdz_), 7.40 (td, *J* = 7.5/1.1 Hz, 1H, 8‐*H*_bdz_), 7.45 – 7.54 (m, 5H, 2‐*H*_Phe_, 3‐*H*_Phe_, 4‐*H*_Phe_, 5‐*H*_Phe_, 6‐*H*_Phe_), 7.74 (ddd, *J* = 8.5/7.3/1.6 Hz, 1H, 7‐*H*_bdz_), 7.86 (d, *J* = 8.4 Hz, 1H, 6‐*H*_bdz_), 9.21 (s, 1H, O*H*), 9.36 (d, *J* = 8.0 Hz, 1H, N*H*). ^13^C NMR (151 MHz, DMSO‐d6): δ (ppm) = 41.0 (NHCO*C*H_2_), 46.0 (q, *J* = 32.6 Hz, N*C*H_2_CF_3_), 67.6 (C‐3_bdz_), 114.9 (2C, C‐3_Phenol_, C‐5_Phenol_), 123.2 (C‐6_bdz_), 124.3 (q, *J* = 281.4 Hz, NCH_2_ *C*F_3_), 125.9 (C‐8_bdz_), 126.2 (C‐1_Phenol_), 128.5 (2 C, C‐3_Phe_, C‐5_Phe_), 128.9 (2 C, C‐2_Phe_, C‐6_Phe_), 129.4 (C‐9_bdz_), 129.8 (C‐5a_bdz_), 130.0 (2C, C‐2_Phenol_, C‐6_Phenol_), 130.9 (C‐4_Phe_), 132.4 (C‐7_bdz_), 137.4 (C‐1_Phe_), 140.3 (C‐9a_bdz_), 155.9 (C‐4_Phenol_), 167.2 (C‐5_bdz_), 168.1 (HN*C*═O), 171.1 (C═O_bdz_). IR (neat): ν̃ (cm^−1^) = 3251 (OH, NH), 1686 (C═O, lactam), 1639 (C═O, amide), 1601 (C═C, aryl), 1246 (OH, phenol),1161 (C─N, amide), 1119 (CF_3_). Exact mass (APCI): *m/z* = 468.1498 (calcd. 468.1530 for C_25_H_21_F_3_N_3_O_3_ [M + H]^+^).

#### 4.1.12 General Procedure A for the HBTU Coupling of 3‐aminobenzodiazepin‐2‐one (**9**) With Various Carboxylic Acids

Et_3_N (0.72 mL, 526 mg, 5.2 mmol, 5.2 eq.) was added to a mixture of 3‐aminobenzodiazepin‐2‐one **9** (251 mg, 1 mmol, 1.0 eq.), the respective carboxylic acid (1.5 mmol, 1.5 eq.), and HBTU (834 mg, 2.2 mmol, 2.2 eq.) in CH_2_Cl_2_ (9 mL) and DMF (2 mL). The mixture was stirred overnight. An aqueous solution of citric acid (10%, 100 mL) was added, and the product was extracted with ethyl acetate (3 × 100 mL). The organic layer was dried (Na_2_SO_4_), filtered, and concentrated in vacuo.

#### 4.1.13 Synthesis of 2‐[2,4‐bis(trifluoromethyl)phenyl]‐*N*‐(2‐oxo‐5‐phenyl‐2,3‐dihydro‐*1H*‐1,4‐benzodiazepin‐3‐yl)acetamide (**10a**)


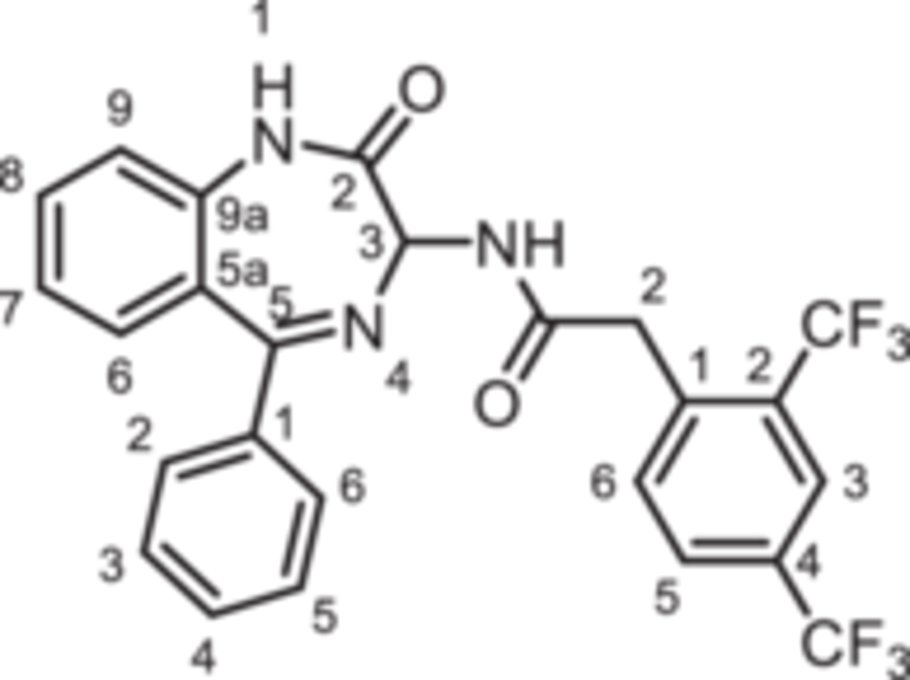


According to the General Procedure A, 3‐aminobenzodiazepin‐2‐one **9** (2.22 g, 8.82 mmol,1.0 eq.) was reacted with HBTU (7.36 g, 19.4 mmol, 2.2 eq.), Et_3_N (6.36 mL, 4.64 g, 45.90 mmol, 5.2 eq.) and 2‐[2,4‐bis(trifluoromethyl)phenyl]acetic acid (2.43 mL, 3.60 g, 13.23 mmol, 1.5 eq.). The residue was purified by flash chromatography (SiO_2_, cyclohexane/ethyl acetate, gradient 100:0 → 0:100). Colorless solid, mp 268°C–269°C, yield 2.18 g (4.32 mmol, 49%). Purity (HPLC): 99.6% (*t*_R_ = 22.1 min). Chemical formula: C_25_H_17_F_6_N_3_O_2_ (505.4 g/mol). TLC: Rf = 0.23 (cyclohexane/ethyl acetate 50:50). ^1^H NMR (600 MHz, DMSO‐d6): *δ* (ppm) = 4.05 (s, 2H, aryl‐C*H*_2_‐C═O), 5.25 (d, *J* = 7.9 Hz, 1H, 3‐*H*_bdz_), 7.24 (ddd, *J* = 8.1/7.1/1.0 Hz, 1H, 9‐*H*_bdz_), 7.27–7.33 (m, 2H, 6‐*H*_bdz_, 7‐*H*_bdz_), 7.42–7.57 (m, 5H, 2‐*H*_Phe_, 3‐*H*_Phe_, 4‐*H*_Phe_, 5‐*H*_Phe_, 6‐*H*_Phe_), 7.63 (ddd, *J* = 8.5/7.2/1.2 Hz, 1H, 8‐*H*_bdz_), 7.80 (d, *J* = 8.1 Hz, 1H, 6‐$H_{{\left({\text{CF}}_{3}\right)}_{2}\text{Phe}}$), 7.97 (s, 1H, 3‐$H_{{\left({\text{CF}}_{3}\right)}_{2}\text{Phe}}$), 8.04 (d, *J* = 8.2 Hz, 1H, 5‐$H_{{\left({\text{CF}}_{3}\right)}_{2}\text{Phe}}$), 9.54 (d, *J* = 8.2 Hz, 1H, N*H*_acetyl_), 10.87 (s, 1H, N*H*_bdz_). ^13^C NMR (151 MHz, DMSO‐d6): *δ* (ppm) = 38.4 (aryl‐*C*H_2_‐C═O), 68.2 (C‐3_bdz_), 121.4 (C‐6_bdz_), 122.4 (m, C‐$3_{{\left({\text{CF}}_{3}\right)}_{2}\text{Phe}}$), 122.6 (q, *J* = 274.2 Hz, *C*F_3_‐C$4_{{\left({\text{CF}}_{3}\right)}_{2}\text{Phe}}$), 123.3 (C‐9_bdz_), 124.4 (q, *J* = 282.7 Hz, *C*F_3_‐C$2_{{\left({\text{CF}}_{3}\right)}_{2}\text{Phe}}$), 126.3 (C‐5a_bdz_), 127.8 (q, *J* = 32.8 Hz, C‐$4_{{\left({\text{CF}}_{3}\right)}_{2}\text{Phe}}$), 128.3 (2 C, C‐3_Phe_, C‐5_Phe_), 128.6 (q, *J* = 30.6 Hz, C‐$2_{{\left({\text{CF}}_{3}\right)}_{2}\text{Phe}}$), 128.9 (q, *J* = 4.0 Hz, C‐$5_{{\left({\text{CF}}_{3}\right)}_{2}\text{Phe}}$), 129.4 (2 C, C‐2_Phe_, C‐6_Phe_), 130.4 (C‐7_bdz_), 130.6 (C‐4_Phe_), 132.2 (C‐8_bdz_), 134.4 (C‐$6_{{\left({\text{CF}}_{3}\right)}_{2}\text{Phe}}$), 138.1 (C‐1_Phe_), 138.8 (C‐9a_bdz_), 139.4 (C‐$1_{{\left({\text{CF}}_{3}\right)}_{2}\text{Phe}}$), 167.0 (C‐5_bdz_), 167.7 (C═O_acetyl_), 168.8 (C═O_bdz_). IR (neat): ν̃ (cm^−1^) = 3051 (C–H, aryl), 1670 (C═O, amide/lactam), 1605 (C═C, aryl), 1161 (C─N, amide, CF_3_). Exact mass (APCI): *m/z* = 506.1266 (calcd. 506.1298 for C_25_H_18_F_6_N_3_O_2_ [M + H]^+^).

#### 4.1.14 Synthesis of 2‐(2‐bromphenyl)‐*N*‐(2‐oxo‐5‐phenyl‐2,3‐dihydro‐*1H*‐1,4‐benzodiazepin‐3‐yl)acetamide (**10b**)


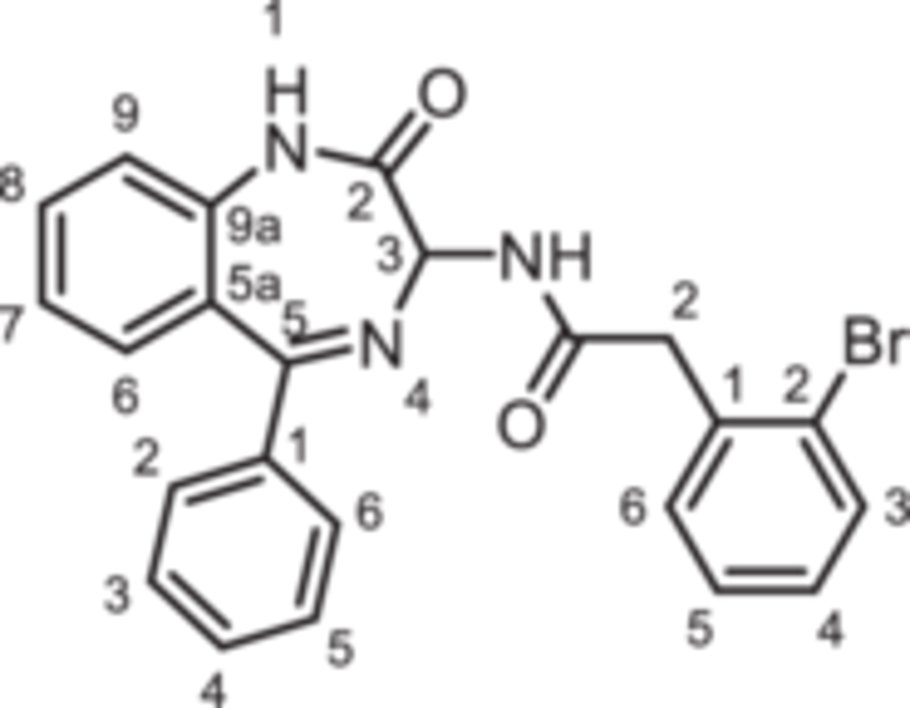


According to the General Procedure A, 3‐aminobenzodiazepin‐2‐one **9** (452 mg, 1.80 mmol,1.0 eq.) was reacted with HBTU (1.50 g, 3.96 mmol, 2.2 eq.), Et_3_N (1.30 mL, 947 mg, 9.36 mmol, 5.2 eq.) and 2‐(2‐bromophenyl)acetic acid (581 mg, 2.70 mmol, 1.5 eq.). The residue was purified by recrystallization from MeOH. Colorless solid, mp 272°C–273°C, yield 487 mg (1.09 mmol, 54%). Purity (HPLC): 100.0% (tR = 19.8 min). Chemical formula: C_23_H_18_BrN_3_O_2_ (448.3 g/mol). TLC: Rf = 0.19 (cyclohexane/ethyl acetate 50:50). ^1^H NMR (600 MHz, DMSO‐d6): *δ* (ppm) = 3.84 (d, *J* = 2.6 Hz, 2H, aryl‐C*H*_2_‐C═O), 5.27 (d, *J* = 7.9 Hz, 1H, 3‐*H*_bdz_), 7.18 (td, *J* = 7.7/1.8 Hz, 1H, 8‐*H*_bdz_), 7.24 (ddd, *J* = 8.0/7.2/1.1 Hz, 1H, 9‐*H*_bdz_), 7.28–7.36 (m, 3H, 6‐*H*_bdz_, 7‐*H*_bdz_, 5‐*H*_BrPhe_), 7.42–7.55 (m, 6H, 2‐*H*_Phe_, 3‐*H*_Phe_, 4‐*H*_Phe_, 5‐*H*_Phe_, 6‐*H*_Phe,_ 6‐*H*_BrPhe_), 7.59 (dd, *J* = 7.9/1.2 Hz, 1H, 3‐*H*_BrPhe_), 7.63 (ddd, *J* = 8.4/7.2/1.5 Hz, 1H, 4‐*H*_BrPhe_), 9.33 (d, *J* = 8.0 Hz, 1H, N*H*_acetyl_), 10.86 (s, 1H, N*H*_bdz_). ^13^C NMR (151 MHz, DMSO‐d6): *δ* (ppm) = 42.0 (aryl‐*C*H_2_‐C═O), 68.0 (C‐3_bdz_), 121.4 (C‐5_BrPhe_), 123.3 (C‐9_bdz_), 124.5 (C‐2_BrPhe_), 126.3 (C‐5a_bdz_), 127.5 (C‐6_bdz_), 128.3 (2 C, C‐3_Phe_, C‐5_Phe_), 128.6 (C‐8_bdz_), 129.4 (2 C, C‐2_Phe_, C‐6_Phe_), 130.4 (C‐7_bdz_), 130.6 (C‐4_Phe_), 131.7 (C‐6_BrPhe_), 132.1 (C‐4_BrPhe_), 132.2 (C‐3_BrPhe_), 135.9 (C‐1_BrPhe_), 138.1 (C‐1_Phe_), 138.8 (C‐9a_bdz_), 166.9 (C‐5_bdz_), 167.8 (C═O_bdz_), 169.3(C═O_acetyl_). IR (neat): ν̃ (cm^−1^) = 3052 (C–H, aryl), 1678 (C═O, amide/lactam), 1601 (C═C, aryl), 1153 (C–N, amide). Exact mass (APCI): *m/z* = 448.0638 (calcd. 448.0655 for C_23_H_19_^79^BrN_3_O_2_ [M + H]^+^).

#### 4.1.15 Synthesis of 3‐bromo‐5‐iodo‐*N*‐(2‐oxo‐5‐phenyl‐2,3‐dihydro‐1*H*‐benzodiazepin‐3‐yl)benzamide (**10c**)


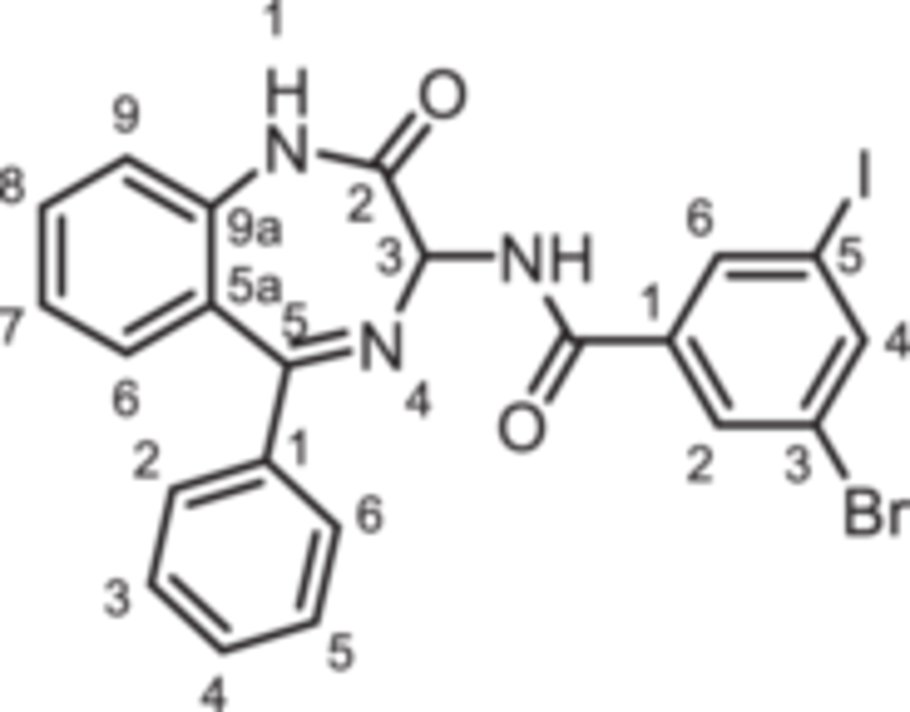


According to the General Procedure A, 3‐aminobenzodiazepin‐2‐one **9** (452 mg, 1.80 mmol, 1.0 eq.) was reacted with HBTU (1.50 g, 3.96 mmol, 2.2 eq.), Et_3_N (1.30 mL, 947 mg, 9.36 mmol, 5.2 eq.) and 3‐bromo‐5‐iodobenzoic acid (883 mg, 2.70 mmol, 1.5 eq.). The residue was purified by recrystallization from MeOH. Orange solid, mp 275°C –276°C, yield 786 mg (1.40 mmol, 78%). Purity (HPLC): 98.4% (*t*_R_ = 22.8 min). Chemical formula: C_22_H_15_BrIN_3_O_2_ (560.2 g/mol). TLC: R_f_ = 0.38 (cyclohexane/ethyl acetate 60:40). ^1^H NMR (600 MHz, DMSO‐d_6_): *δ* (ppm) = 5.46 (d, *J* = 7.6 Hz, 1H, 3‐*H*_bdz_), 7.26 (t, *J* = 7.7 Hz, 1H, 7‐*H*_bdz_), 7.30–7.37 (m, 2H, 6‐*H*_bdz_, 9‐*H*_bdz_), 7.43–7.49 (m, 2H, 3‐*H*_Phe_, 5‐*H*_Phe_), 7.50–7.57 (m, 3H, 2‐*H*_Phe_, 4‐*H*_Phe_, 6‐*H*_Phe_), 7.66 (ddd, *J* = 8.4/7.2/1.6 Hz, 1H, 8‐*H*_bdz_), 8.17 (t, *J* = 1.7 Hz, 1H, 4‐*H*_IBrPhe_), 8.21 (t, *J* = 1.6 Hz, 1H, 2‐*H*_IBrPhe_), 8.38 (t, *J* = 1.6 Hz, 1H, 6‐*H*_IBrPhe_), 9.91 (d, *J* = 7.5 Hz, 1H, N*H*_bdz_), 10.93 (s, 1H, N*H*_benzamide_). ^13^C NMR (151 MHz, DMSO‐d_6_): *δ* (ppm) = 69.3 (C‐3_bdz_), 95.7 (C‐5_IBrPhe_), 121.4 (C‐9_bdz_), 122.4 (C‐3_IBrPhe_), 123.3 (C‐7_bdz_), 126.2 (C‐5a_bdz_), 128.3 (2 C, C‐3_Phe_, C‐5_Phe_), 129.5 (2 C, C‐2_Phe_, C‐6_Phe_), 130.1 (C‐2_IBrPhe_), 130.5 (C‐4_Phe_), 130.6 (C‐6_bdz_), 132.2 (C‐8_bdz_), 135.4 (C‐6_IBrPhe_), 136.9 (C‐1 _IBrPhe_), 138.1 (C‐1_Phe_), 138.8 (C‐9a_bdz_), 141.6 (C‐4_IBrPhe_), 163.6 (C═O_benzamide_), 167.3 (C‐5_bdz_), 167.8 (C═CO_bdz_). IR (neat): ν̃ (cm^−1^) = 3075 (C─H, aryl), 1662 (C═O, amide/lactam), 1605 (C═C, aryl), 1172 (C─N, amide). Exact mass (APCI): *m/z* = 559.9448 (calcd. 559.9465 for C_22_H_16_^79^BrIN_3_O_2_ [M + H]^+^).

#### 4.1.16 Synthesis of *N*‐[2‐oxo‐5‐phenyl‐2,3‐dihydro‐*1H*‐1,4‐benzodiazepin‐3‐yl]‐2‐(pyridin‐3‐yl)acetamide (**10d**)


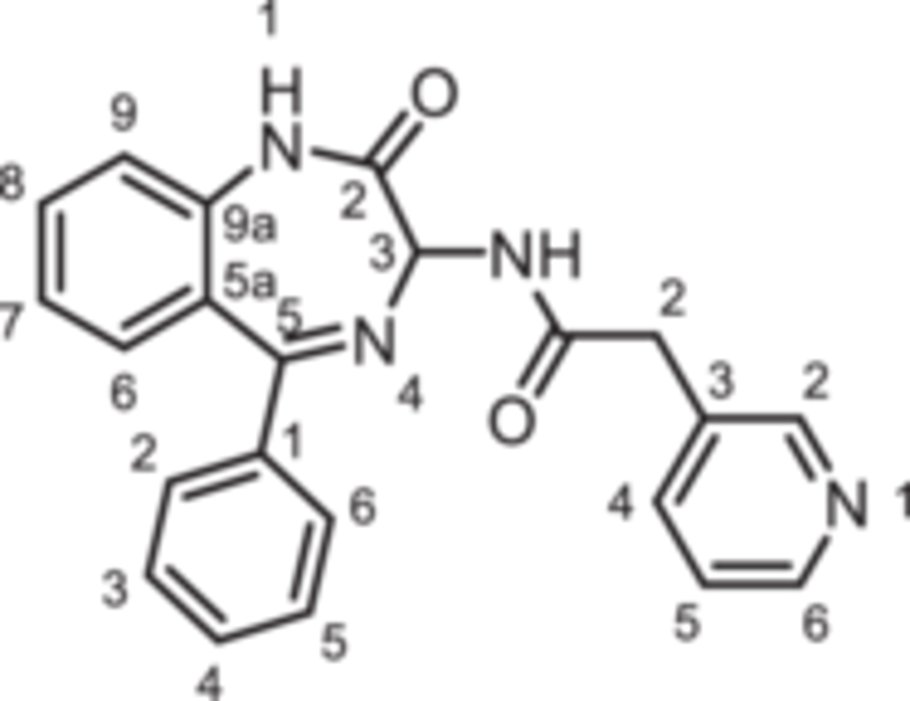


According to the General Procedure A, 3‐aminobenzodiazepin‐2‐one **9** (452 mg, 1.80 mmol, 1.0 eq.) was reacted with HBTU (1.50 g, 3.96 mmol, 2.2 eq.), Et_3_N (1.30 mL, 947 mg, 9.36 mmol, 5.2 eq.) and 2‐(pyridin‐3‐yl)acetic acid (370 mg, 2.70 mmol, 1.5 eq.). The residue was purified by flash chromatography (SiO_2_, CH_2_Cl_2_/MeOH, gradient 100:0 → 85:15). Colorless solid, mp 242°C–243°C, yield 87 mg (0.23 mmol, 13%). Purity (HPLC): 99.1% (*t*_R_ = 14.0 min). Chemical formula: C_22_H_18_N_4_O_2_ (370.4 g/mol). TLC: R_f_ = 0.18 (CH_2_Cl_2_/MeOH 100:4.5). ^1^H NMR (600 MHz, DMSO‐d_6_): *δ* (ppm) = 3.71 (d, *J* = 7.3 Hz, 2H, aryl‐C*H*_2_–C═O), 5.23 (d, *J* = 7.9 Hz, 1H, 3‐*H*_bdz_), 7.24 (t, *J* = 7.5 Hz, 1H, 7‐*H*_bdz_), 7.27–7.35 (m, 3H, 6‐*H*_bdz_, 9‐*H*_bdz_, 5‐*H*_pyr_), 7.42–7.56 (m, 5H, 2‐*H*_Phe_, 3‐*H*_Phe_, 4‐*H*_Phe_, 5‐*H*_Phe_, 6‐*H*_Phe_), 7.63 (ddd, *J* = 8.3/7.1/1.6 Hz, 1H, 8‐*H*_bdz_), 7.74 (dt, *J* = 7.8/2.0 Hz, 1H, 4‐*H*_pyr_), 8.44 (dd, *J* = 4.8/1.7 Hz, 1H, 6‐*H*_pyr_), 8.52 (d, *J* = 1.8 Hz, 1H, 2‐*H*_pyr_), 9.46 (d, *J* = 7.9 Hz, 1H, N*H*_acetyl_), 10.85 (s, 1H, N*H*_bdz_). ^13^C NMR (151 MHz, DMSO‐d_6_): *δ* (ppm) = 38.8 (aryl‐*C*H_2_‐C═O), 68.2 (C‐3_bdz_), 121.4 (C‐9_bdz_), 123.2 (C‐5_pyr_), 123.3 (C‐7_bdz_), 126.3 (C‐5a_bdz_), 128.3 (2 C, C‐3_Phe_, C‐5_Phe_), 129.4 (2 C, C‐2_Phe_, C‐6_Phe_), 130.4 (C‐4_Phe_), 130.6 (C‐6_bdz_), 131.9 (C‐3_pyr_), 132.1 (C‐8_bdz_), 136.6 (C‐4_pyr_), 138.1 (C‐1_Phe_), 138.8 (C‐9a_bdz_), 147.6 (C‐6_pyr_), 150.1 (C‐2_pyr_), 167.0 (C‐5_bdz_), 167.8 (C═O_bdz_), 170.0 (C═O_acetyl_). IR (neat): ν̃ (cm^−1^) = 3051 (C─H, aryl), 1697 (C═O, lactam), 1639 (C═O, amide), 1612 (C═C, aryl), 1180 (C─N, amide). Exact mass (APCI): *m/z* = 371.1492 (calcd. 371.1503 for C_22_H_19_N_4_O_2_ [M + H]^+^).

#### 4.1.17 Synthesis of *N*‐(2‐oxo‐5‐phenyl‐2,3‐dihydro‐*1H*‐1,4‐benzodiazepin‐3‐yl)‐3‐nitrobenzamide (**10e**)


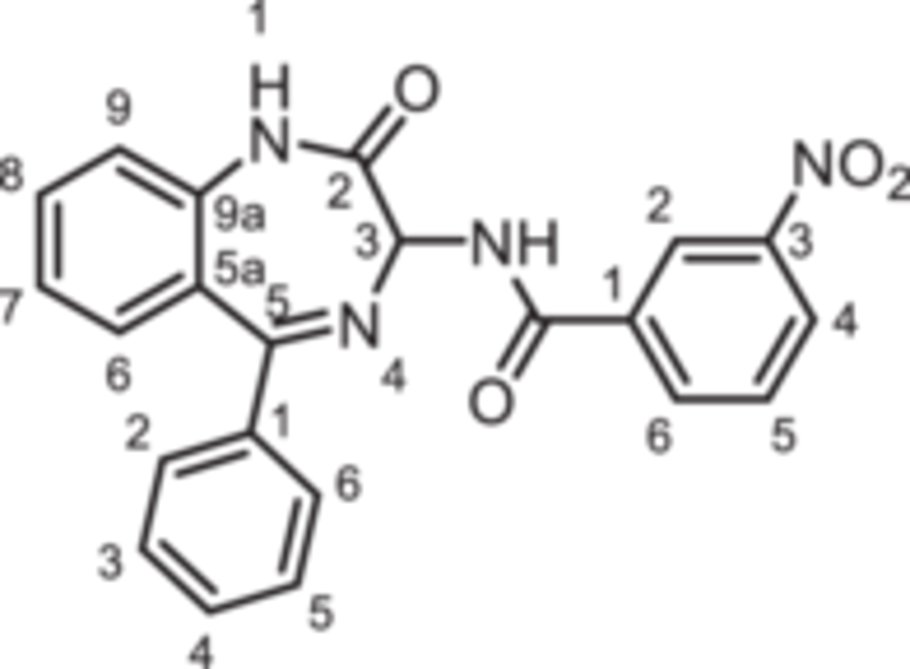


At 0°C a mixture of 3‐aminobenzodiazepine **9** (503 mg, 2.0 mmol, 1.0 eq.) and Et_3_N (0.42 mL, 304 mg, 3.0 mmol, 1.5 eq.) was stirred in THF (2 mL) and DMF (1 mL) for 15 min. Then, a solution of 3‐nitrobenzoyl chloride (557 mg, 3.0 mmol, 1.5 eq.) in THF (4 mL) and DMF (1 mL) was added dropwise over 15 min. The ice bath was removed, and the mixture was stirred over night at room temperature. An aqueous solution of citric acid (10%, 100 mL) was added, and the product was extracted with ethyl acetate (3 × 100 mL). The organic layer was dried (Na_2_SO_4_), filtered, and concentrated in vacuo. The residue was purified by recrystallization from MeOH. Slightly yellow solid, mp 267°C–268°C, yield 545 mg (1.36 mmol, 68%). Purity (HPLC): 99.3% (*t*_R_ = 19.5 min). Chemical formula: C_22_H_16_N_4_O_4_ (400.4 g/mol). TLC: Rf = 0.18 (cyclohexane/ethyl acetate 50:50). ^1^H NMR (600 MHz, DMSO‐d6): *δ* (ppm) = 5.52 (d, *J* = 7.5 Hz, 1H, 3‐*H*_bdz_), 7.27 (td, *J* = 7.6/1.1 Hz, 1H, 7‐*H*_bdz_), 7.32–7.39 (m, 2H, 6‐*H*_bdz_, 9‐*H*_bdz_), 7.44–7.50 (m, 2H, 3‐*H*_Phe_, 5‐*H*_Phe_), 7.51–7.58 (m, 3H, 2‐*H*_Phe_, 4‐*H*_Phe_, 6‐*H*_Phe_), 7.67 (ddd, *J* = 8.3/7.2/1.6 Hz, 1H, 8‐*H*_bdz_), 7.82 (t, *J* = 8.0 Hz, 1H, 5‐*H*_NO2Phe_), 8.42–8.49 (m, 2H, 4‐*H*_NO2Phe_, 6‐*H*_NO2Phe_), 8.86 (t, *J* = 2.0 Hz, 1H, 2‐*H*_NO2Phe_), 10.10 (d, *J* = 7.6 Hz, 1H, N*H*_benzamide_), 10.95 (s, 1H, N*H*_bdz_). ^13^C NMR (151 MHz, DMSO‐d6): *δ* (ppm) = 69.4 (C‐3_bdz_), 121.4 (C‐9_bdz_), 122.7 (C‐2_NO2Phe_), 123.3 (C‐7_bdz_), 126.2 (C‐4_NO2Phe_), 126.3 (C‐5a_bdz_), 128.3 (2 C, C‐3_Phe_, C‐5_Phe_), 129.5 (2 C, C‐2_Phe_, C‐6_Phe_), 130.1 (C‐5_NO2Phe_), 130.5 (C‐4_Phe_), 130.6 (C‐6_bdz_), 132.3 (C‐8_bdz_), 134.2 (C‐6_NO2Phe_), 135.1 (C‐1_NO2Phe_), 138.1 (C‐1_Phe_), 138.7 (C‐9a_bdz_), 147.7 (C‐3_NO2Phe_), 164.5 (C═O_benzamide_), 167.3 (C‐5_bdz_), 167.8 (C═O_bdz_). IR (neat): ν̃ (cm^–1^) = 3055 (C–H, aryl), 1689 (C═O, lactam), 1651 (C═O, benzamide), 1598 (C═C, aryl), 1516 (NO_2_), 1165 (C–N, amide). Exact mass (APCI): *m/z* = 401.1230 (calcd. 401.1244 for C_22_H_17_N_4_O_4_ [M + H]^+^).

#### 4.1.18 Synthesis of 2‐[2,4‐bis(trifluoromethyl)phenyl]‐*N*‐[1‐heptyl‐2‐oxo‐5‐phenyl‐2,3‐dihydro‐1*H*‐1,4‐benzodiazepin‐3‐yl]acetamide (**12a**)


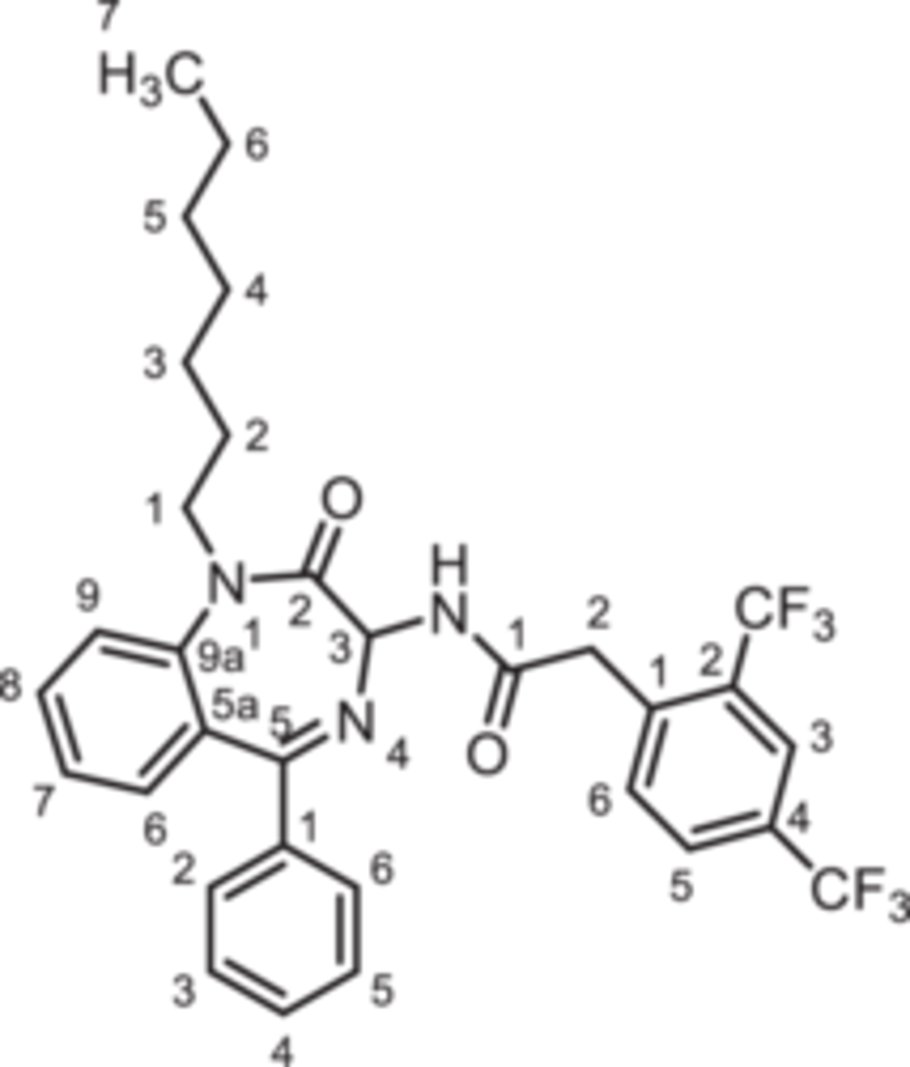


At 0°C, 1,4‐benzodiazepine **10a** (101 mg, 0.20 mmol, 1.00 eq.) was dissolved in THF (0.7 mL) and DMF (0.3 mL) and was then deprotonated with NaH (60% dispersion in paraffine, 10 mg, 0.24 mmol, 1.20 eq.). The ice bath was removed, and heptyl tosylate (**11a**, 111 mg, 0.41 mmol, 2.05 eq.) was added. Stirring of the mixture was continued at 50°C for 3 h. The mixture was then poured into a saturated solution of NH_4_Cl (10 mL). CH_2_Cl_2_ (30 mL) and H_2_O (20 mL) were added, and the product was extracted with CH_2_Cl_2_ (3 × 30 mL). The combined organic layers were dried (Na_2_SO_4_), filtered, and concentrated in vacuo. The residue was purified by flash chromatography (SiO_2_, CH_2_Cl_2_/CH_3_OH, gradient 100:0 → 90:10). Colorless solid, mp 197°C– 198°C, yield 37 mg (0.06 mmol, 31%). Purity (HPLC): 99.5% (*t*_R_ = 27.3 min). Chemical formula: C_32_H_31_F_6_N_3_O_2_ (603.6 g/mol). TLC: *R*_f_ = 0.32 (cyclohexane/ethyl acetate 70:30). ^1^H NMR (600 MHz, DMSO‐d_6_): *δ* (ppm) = 0.67 (t, *J* = 7.3 Hz, 3H, C*H*_3heptyl_), 0.79–1.09 (m, 8H, 3‐*H*_heptyl_, 4‐*H*_heptyl_, 5‐*H*_heptyl_, 6‐*H*_heptyl_), 1.20–1.27 (m, 1H, 2‐*H*_heptyl_), 1.35–1.44 (m, 1H, 2‐*H*_heptyl_), 3.75 (dt, *J* = 13.9/5.6 Hz, 1H, 1‐*H*_heptyl_), 4.03 (s, 2H, aryl‐*C*H_2_‐C═O), 4.35 (dt, *J* = 13.8/6.1 Hz, 1H, 1‐*H*_heptyl_), 5.31 (d, *J* = 7.9 Hz, 1H, 3‐*H*_bdz_), 7.30–7.38 (m, 2H, 8‐*H*_bdz_, 9‐*H*_bdz_), 7.45–7.58 (m, 5H, 2‐*H*_Phe_, 3‐*H*_Phe_, 4‐*H*_Phe_, 5‐*H*_Phe_, 6‐*H*_Phe_), 7.67–7.75 (m, 3H, 6‐*H*_bdz_, 7‐*H*_bdz_, 6‐$H_{{\left({\text{CF}}_{3}\right)}_{2}\text{Phe}}$), 7.96 (s, 1H, 3‐$H_{{\left({\text{CF}}_{3}\right)}_{2}\text{Phe}}$), 8.03 (d, *J* = 8.2 Hz, 1H, 5‐$H_{{\left({\text{CF}}_{3}\right)}_{2}\text{Phe}}$), 9.58 (d, *J* = 7.9 Hz, 1H, N*H*). ^13^C NMR (151 MHz, DMSO‐d_6_): *δ* (ppm) = 13.8 (*C*H_3heptyl_), 21.8 (C‐6_heptyl_), 25.6 (C‐3_heptyl_), 27.1 (C‐2_heptyl_), 28.2 (C‐4_heptyl_), 31.0 (C‐5_heptyl_), 38.3 (aryl‐*C*H_2_‐C═O), 45.5 (C‐1_heptyl_), 68.1 (C‐3_bdz_), 122.4 (m, C‐$3_{{\left({\text{CF}}_{3}\right)}_{2}\text{Phe}}$), 122.9 (q, *J* = 273.9 Hz, *C*F_3_‐C$4_{{\left({\text{CF}}_{3}\right)}_{2}\text{Phe}}$), 123.0 (C‐$6_{{\left({\text{CF}}_{3}\right)}_{2}\text{Phe}}$), 125.0 (C‐8_bdz_), 125.2 (q, *J* = 283.3 Hz, *C*F_3_‐C$2_{{\left({\text{CF}}_{3}\right)}_{2}\text{Phe}}$), 127.9 (q, *J* = 32.9 Hz, C‐$4_{{\left({\text{CF}}_{3}\right)}_{2}\text{Phe}}$), 128.4 (2 C, C‐3_Phe_, C‐5_Phe_), 128.6 (q, *J* = 30.6 Hz, C‐$2_{{\left({\text{CF}}_{3}\right)}_{2}\text{Phe}}$), 128.9 (q, *J* = 3.5 Hz, C‐$5_{{\left({\text{CF}}_{3}\right)}_{2}\text{Phe}}$), 129.1 (2 C, C‐2_Phe_, C‐6_Phe_), 129.5 (C‐9_bdz_), 129.6 (C‐5a_bdz_), 130.8 (C‐4_Phe_), 132.2 (C‐6_bdz_), 134.4 (C‐7_bdz_), 137.3 (C‐1_Phe_), 139.4 (C‐$1_{{\left({\text{CF}}_{3}\right)}_{2}\text{Phe}}$), 141.0 (C‐9a_bdz_), 166.5 (C‐5_bdz_), 167.0 (C═O_bdz_), 168.7 (C═O_acetyl_). IR (neat): ν̃ (cm^–1^) = 3244 (NH, amide), 2932 and 2859 (CH_2_), 1681 (C═O, lactam), 1651 (C═O, amide), 1601 (C═C, aryl), 1165 (C─N, amide), 1122 (CF_3_). Exact mass (APCI): *m/z* = 604.2424 (calcd. 604.2393 for C_32_H_32_F_6_N_3_O_2_ [M + H]^+^).

#### 4.1.19 Synthesis of 2‐[2,4‐bis(trifluoromethyl)phenyl]‐*N*‐[1‐(2‐cyclohexylethyl)‐2‐oxo‐5‐phenyl‐2,3‐dihydro‐1*H*‐1,4‐benzodiazepin‐3‐yl]acetamide (**12b**)


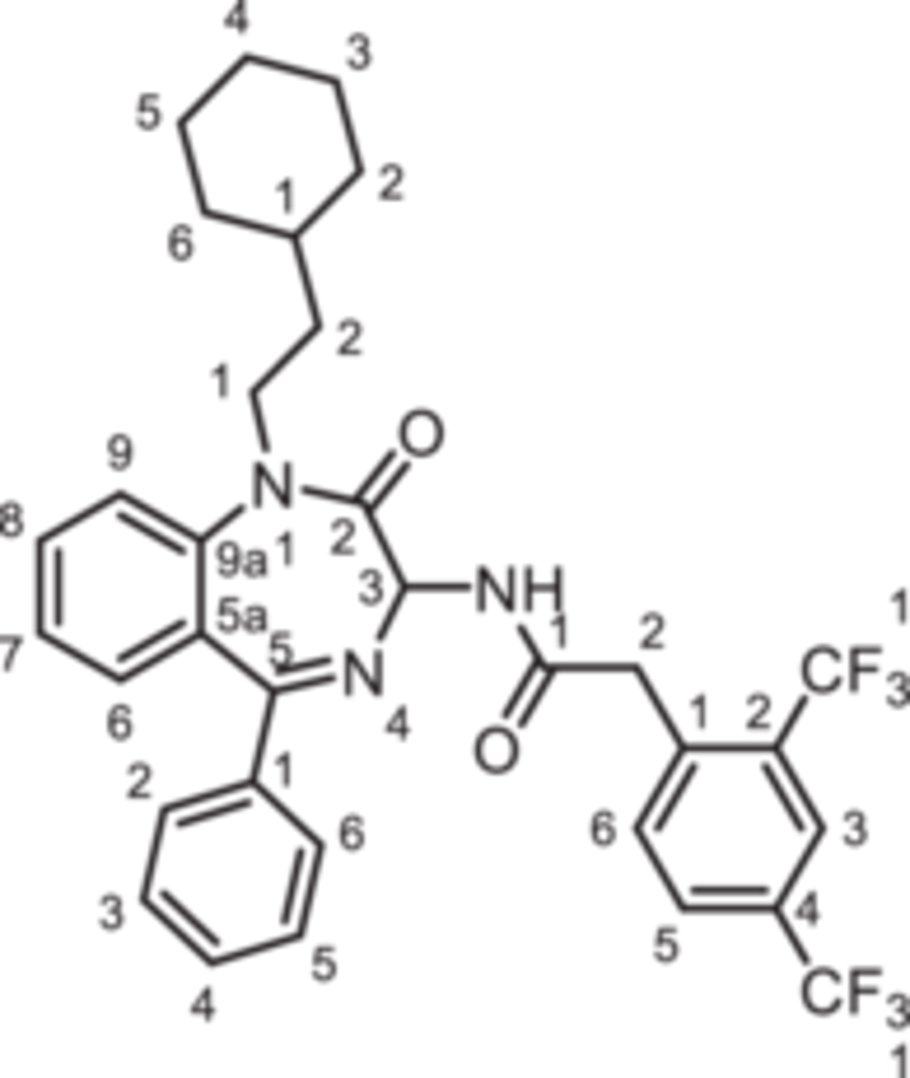


At 0°C, 1,4‐benzodiazepine **10a** (101 mg, 0.20 mmol, 1.00 eq.) in DMF (0.9 mL) was deprotonated with NaH (60% dispersion in paraffine, 10 mg, 0.24 mmol, 1.20 eq.). The ice bath was removed and (2‐cyclohexylethyl) tosylate (**11b**, 115 mg, 0.28 mmol, 1.40 eq.) was added. Stirring of the mixture was continued at 40°C for 2 h. The mixture was then poured into a saturated solution of NH_4_Cl (10 mL). CH_2_Cl_2_ (30 mL) and H_2_O (20 mL) were added, and the product was extracted with CH_2_Cl_2_ (3 × 30 mL). The combined organic layers were dried (Na_2_SO_4_), filtered, and concentrated in vacuo. The residue was purified by flash chromatography (SiO_2_, CH_2_Cl_2_/CH_3_OH, gradient 100:0 → 90:10). Colorless solid, mp 202°C–203°C, yield 41 mg (0.07 mmol, 33%). Purity (HPLC): 99.7% (*t*_R_ = 27.2 min). Chemical formula: C_33_H_31_F_6_N_3_O_2_ (615.6 g/mol). TLC: *R*_f_ = 0.31 (cyclohexane/ethyl acetate 70:30). ^1^H NMR (600 MHz, DMSO‐d_6_): *δ* (ppm) = 0.57–0.79 (m, 5H, 2‐*H*_ax. cyclohexyl_, 3‐*H*_ax. cyclohexyl_, 4‐*H*_ax. cyclohexyl_, 5‐*H*_ax. cyclohexyl_, 6‐*H*_ax. cyclohexyl_), 1.15–1.28 (m, 3H, NCH_2_C*H*_2_cyclohexyl, 1‐*H*_cyclohexyl_), 1.33– 1.47 (m, 4H, 2‐*H*_eq. cyclohexyl_, 3‐*H*_eq. cyclohexyl,_ 5‐*H*_eq. cyclohexyl_, 6‐*H*_eq. cyclohexyl_), 1.52–1.62 (m, 1H, 4‐*H*_eq. cyclohexyl_), 3.76 (dt, *J* = 14.1/5.4 Hz, 1H, NC*H*_2_CH_2_cyclohexyl), 4.02 (s, 2H, aryl‐C*H*_2_‐C═O), 4.45 (dt, *J* = 14.1/7.7 Hz, 1H, NC*H*_2_CH_2_cyclohexyl), 5.31 (d, *J* = 8.0 Hz, 1H, 3‐*H*_bdz_), 7.32–7.39 (m, 2H, 8‐*H*_bdz_, 9‐*H*_bdz_), 7.46–7.60 (m, 5H, 2‐*H*_Phe_, 3‐*H*_Phe_, 4‐*H*_Phe_, 5‐*H*_Phe_, 6‐*H*_Phe_), 7.68–7.79 (m, 3H, 6‐*H*_bdz_, 7‐*H*_bdz_, 6‐$H_{{\left({\text{CF}}_{3}\right)}_{2}\text{Phe}}$), 7.96 (s, 1H, 3‐$H_{{\left({\text{CF}}_{3}\right)}_{2}\text{Phe}}$), 8.03 (d, *J* = 8.1 Hz, 1H, 5‐$H_{{\left({\text{CF}}_{3}\right)}_{2}\text{Phe}}$), 9.62 (d, *J* = 8.0 Hz, 1H, N*H*). ^13^C NMR (151 MHz, DMSO‐d_6_): *δ* (ppm) = 27.7 (2 C, C‐3_cyclohexyl_, C‐5_cyclohexyl_), 31.7 (C‐4_cyclohexyl_), 33.0 (NCH_2_ *C*H_2_cyclohexyl), 33.4 (2C, C‐2_cyclohexyl_, C‐6_cyclohexyl_), 34.4 (C‐1_cyclohexyl_), 38.3 (aryl‐*C*H_2_‐C═O), 42.5 (N*C*H_2_CH_2_cyclohexyl), 68.0 (C‐3_bdz_), 122.4 (m, C‐$3_{{\left({\text{CF}}_{3}\right)}_{2}\text{Phe}}$), 123.1 (C‐$6_{{\left({\text{CF}}_{3}\right)}_{2}\text{Phe}}$), 123.5 (q, *J* = 274.2, *C*F_3_‐C$4_{{\left({\text{CF}}_{3}\right)}_{2}\text{Phe}}$), 125.0 (C‐8_bdz_), 125.2 (q, *J* = 271.3 *C*F_3_‐C$2_{{\left({\text{CF}}_{3}\right)}_{2}\text{Phe}}$), 127.9 (q, *J* = 32.8 Hz, C‐$4_{{\left({\text{CF}}_{3}\right)}_{2}\text{Phe}}$), 128.4 (2 C, C‐3_Phe_, C‐5_Phe_), 128.6 (q, *J* = 30.6 Hz, C‐$2_{{\left({\text{CF}}_{3}\right)}_{2}\text{Phe}}$), 128.9 (q, *J* = 3.7 Hz, C‐$5_{{\left({\text{CF}}_{3}\right)}_{2}\text{Phe}}$), 129.1 (2 C, C‐2_Phe_, C‐6_Phe_), 129.5 (C‐9_bdz_), 129.6 (C‐5a_bdz_), 130.8 (C‐4_Phe_), 132.1 (C‐6_bdz_), 134.4 (C‐7_bdz_), 137.1 (C‐1_Phe_), 139.4 (C‐$1_{{\left({\text{CF}}_{3}\right)}_{2}\text{Phe}}$), 140.7 (C‐9a_bdz_), 166.3 (C‐5_bdz_), 167.0 (C═O_bdz_), 168.7 (C═O_acetyl_). IR (neat): ν̃ (cm^−1^) = 3248 (NH, amide), 2924 and 2851 (CH_2_), 1681 (C═O, lactam), 1647 (C═O, amide), 1609 (C═C, aryl), 1161 (C–N, amide), 1120 (CF_3_). Exact mass (APCI): *m/z* = 616.2418 (calcd. 616.2393 for C_33_H_32_F_6_N_3_O_2_ [M + H]^+^).

#### 4.1.20 Synthesis of 2‐[2,4‐bis(trifluoromethyl)phenyl]‐*N*‐[1‐(3‐fluoropropyl)‐2‐oxo‐5‐phenyl‐2,3‐dihydro‐1*H*‐1,4‐benzodiazepin‐3‐yl]acetamide (**12c**)


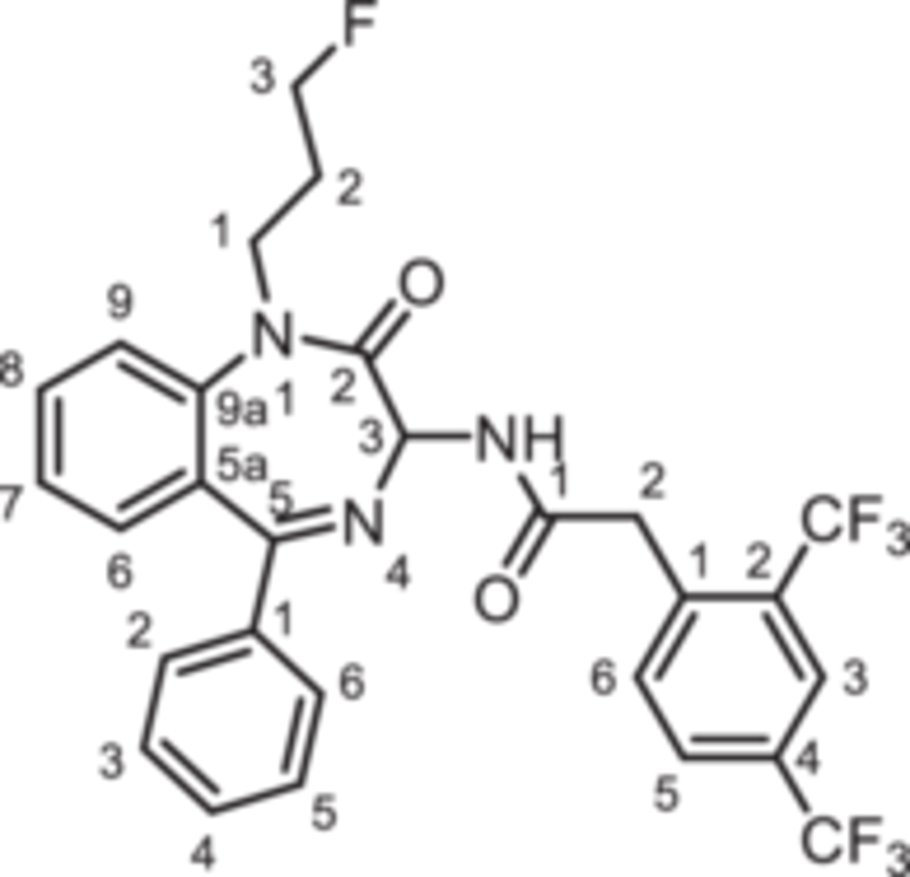


At 0°C, 1,4‐benzodiazepine **10a** (253 mg, 0.50 mmol, 1.00 eq) dissolved in DMF (2.3 mL) was deprotonated with NaH (60% dispersion in paraffine, 21 mg, 0.53 mmol, 1.06 eq.). After stirring for 1 h, the ice bath was removed and (3‐fluoropropyl) tosylate (**11c**, 120 µL, 139 mg, 0.60 mmol, 1.20 eq.) was added. The mixture was heated to 40°C for 1.5 h. Then, the mixture was poured into a saturated solution of NH_4_Cl (20 mL). CH_2_Cl_2_ (50 mL) and H_2_O (30 mL) were added, the layers were separated, and the aqueous layer was extracted with CH_2_Cl_2_ (3 × 50 mL). The combined organic layers were dried (Na_2_SO_4_), filtered, and concentrated in vacuo. The residue was purified by flash chromatography (SiO_2_, cyclohexane/ethyl acetate, gradient 100:0 → 0:100). Colorless solid, mp 181°C–182°C, yield 221 mg (0.39 mmol, 78%). Purity (HPLC): 97.3% (*t*_R_ = 23.7 min). Chemical formula: C_28_H_22_F_7_N_3_O_2_ (565.5 g/mol). TLC: *R*_f_ = 0.22 (cyclohexane/ethyl acetate 60:40). ^1^H NMR (600 MHz, DMSO‐d_6_): *δ* (ppm) = 1.63–1.88 (m, 2H, FCH_2_C*H*_2_CH_2_N), 3.87 (m, 1H, FCH_2_CH_2_C*H*_2_N), 4.03 (s, 2H, aryl‐C*H*_2_‐C═O), 4.14–4.32 (m, 2H, FC*H*_2_CH_2_CH_2_N), 4.40 (m, 1H, FCH_2_CH_2_C*H*_2_N), 5.31 (d, *J* = 7.9 Hz, 1H, 3‐*H*_bdz_), 7.30 (dd, *J* = 7.9/1.5 Hz, 1H, 9‐*H*_bdz_), 7.36 (t, *J* = 7.6 Hz, 1H, 8‐*H*_bdz_), 7.45–7.58 (m, 5H, 2‐*H*_Phe_, 3‐*H*_Phe_, 4‐*H*_Phe_, 5‐*H*_Phe_, 6‐*H*_Phe_), 7.69–7.78 (m, 3H, 6‐*H*_bdz_, 7‐*H*_bdz_, 6‐$H_{{\left({\text{CF}}_{3}\right)}_{2}\text{Phe}}$), 7.97 (s, 1H, 3‐$H_{{\left({\text{CF}}_{3}\right)}_{2}\text{Phe}}$), 8.03 (d, *J* = 8.1 Hz, 1H, 5‐$H_{{\left({\text{CF}}_{3}\right)}_{2}\text{Phe}}$), 9.59 (d, *J* = 7.9 Hz, 1H, N*H*_acetyl_). ^13^C NMR (151 MHz, DMSO‐d_6_): *δ* (ppm) = 28.2 (d, *J* = 19.6 Hz, FCH_2_ *C*H_2_CH_2_N), 38.3, (aryl‐*C*H_2_‐C═O), 43.1 (d, *J* = 5.0 Hz, FCH_2_CH_2_ *C*H_2_N), 68.0 (C‐3_bdz_), 81.1 (d, *J* = 162.9 Hz, F*C*H_2_CH_2_CH_2_N), 122.4 (m, C‐$3_{{\left({\text{CF}}_{3}\right)}_{2}\text{Phe}}$), 122.9 (q, *J* = 279.3 Hz, *C*F_3_‐C$4_{{\left({\text{CF}}_{3}\right)}_{2}\text{Phe}}$), 123.0 (C‐$6_{{\left({\text{CF}}_{3}\right)}_{2}\text{Phe}}$), 124.9 (q, *J* = 279.5 Hz, *C*F_3_‐C$2_{{\left({\text{CF}}_{3}\right)}_{2}\text{Phe}}$), 125.1 (C‐8_bdz_), 127.8 (q, *J* = 32.9 Hz, C‐$4_{{\left({\text{CF}}_{3}\right)}_{2}\text{Phe}}$), 128.5 (2 C, C‐3_Phe_, C‐5_Phe_), 128.6 (q, *J* = 30.7 Hz, C‐$2_{{\left({\text{CF}}_{3}\right)}_{2}\text{Phe}}$), 129.1 (2 C, C‐2_Phe_, C‐6_Phe_), 128.9 (q, *J* = 3.9 Hz, C‐$5_{{\left({\text{CF}}_{3}\right)}_{2}\text{Phe}}$), 129.5 (C‐5a_bdz_), 129.6 (C‐9_bdz_), 130.8 (C‐4_Phe_), 132.3 (C‐6_bdz_), 134.4 (C‐7_bdz_), 137.5 (C‐1_Phe_), 139.4 (C‐$1_{{\left({\text{CF}}_{3}\right)}_{2}\text{Phe}}$), 141.0 (C‐9a_bdz_), 166.7 (C‐5_bdz_), 166.9 (C═O_bdz_), 168.8 (C═Oa_acetyl_). IR (neat): ν̃ (cm^−1^) = 3236 (NH, amide), 1682 (C═O, lactam), 1647 (C═O, amide), 1605 (C═C, aryl), 1173 (C–N, amide), 1110 (CF_3_). Exact mass (APCI): *m/z* = 566.1659 (calcd. 566.1673 for C_28_H_23_F_7_N_3_O_2_ [M + H]^+^).

### 4.2 TEVC Experiments

The human KCNQ1 (GenBank accession number NM_000218) was subcloned into vector pSGEM, linearized by NHE1 digestion, and cRNA was generated using mMessage mMachine kit (Ambion, Austin, TX) according to the manufacturer's instructions as described earlier [32].

Oocyte preparation for TEVC recordings and the conduction of experiments were performed as described by Schreiber et al. [20]. In brief, oocytes were obtained from EcoCyte Bioscience (Dortmund, Germany). Stage IV or V oocytes were injected with 5 ng cRNA encoding K_V_7.1 subunits and stored for 2–4 days at 18°C in Barth's solution containing (in mmol L^−1^): 88 NaCl, 1.1 KCl, 2.4 NaHCO_3_, 0.3 Ca(NO_3_)_2_, 0.3 CaCl_2_, 0.8 MgSO_4_, 15 HEPES‐NaOH, penicillin‐G (31 mg L^−1^), gentamycin (50 mg L^−1^), streptomycin sulfate (20 mg L^−1^), pH 7.6 before electrophysiological recording. Whole‐cell currents in oocytes were recorded at 21°C–22°C by two‐electrode voltage‐clamp (TEVC) using a Turbo Tec 10CD amplifier (NPI Electronic GmbH, Tamm, Germany) and an Ni‐USB‐6221 (National Instruments, Austin, Texas, USA) interface combined with GePulse software (Michael Pusch, Genova, Italy). Recording pipettes pulled from borosilicate glass were filled with 3 M KCl and had resistances of 0.5‐1.5 MΩ. Recordings were performed in ND96 solution containing (in mmol L^−1^) 96 NaCl, 4 KCl, 1 CaCl_2_, 1.8 MgCl_2_, 5 HEPES, pH 7.2–7.4 with 0.1% dimethylsulfoxide. To determine the effects of the synthesized compounds on K_V_7.1 channels, currents were recorded by repetitive 2.5‐second pulses from −80 mV to +40 mV, applied in 20 mV increments, from a holding potential of –80 mV, followed by a 4.5‐s tail pulse at −120 mV. The effect of each compound on K_V_7.1 ion channels is always given as the change of channel activity with the compound in ND96 buffer solution compared with a recording of the same oocyte in ND96 buffer without additional compound. Activity was determined from peak tail currents measured at −120 mV after a pulse to +40 mV.

## Conflicts of Interest

The authors declare no conflicts of interest.

## Supporting Information

The Supporting Information contains some general information used for the synthesis, the procedures for the preparation of tosylates **11a** and **11b**, _1_H and _13_C NMR spectra and HPLC chromatograms of all synthesized compounds.

## Supporting information


Supporting File 1

Supporting File 2

## Data Availability

The data that support the findings of this study are available in the Supporting Information of this article.
