# Supplementary material for: Structure–Activity Relationships of 1,4‐Benzodiazepines Inhibiting the Voltage‐Gated Potassium Channel KV7.1
Source: Arch Pharm (Weinheim). 2026 Oct 23;359(10):e70370. doi: 10.1002/ardp.70370 (PMC13691622; doi:10.1002/ardp.70370)
Supplement: Supplementary file 1 [file ARDP-359-e70370-s001.docx]

**InChi Codes**

**Structure-activity relationship study on 1,4-benzodiazepines inhibiting the**

**voltage-gated potassium channel K_V_7.1**

Florian Roßner,^(a,b)^ Amy Methaq,^(b)^ Guiscard Seebohm,^(a,c)^ * Bernhard Wünsch^(a,b)^ *

| **compd.** | **InChICode** | **activity change [%] ^[a]^** |
| --- | --- | --- |
| ***rac*-1** | InChI=1S/C27H18F9N3O2/c28-25(29,30)14-39-20-9-5-4-8-18(20)22(15-6-2-1-3-7-15)38-23(24(39)41)37-21(40)12-16-10-11-17(26(31,32)33)13-19(16)27(34,35)36/h1-11,13,23H,12,14H2,(H,37,40) | -48 ± 6 ^[b]^ |
|  |  |  |
| ***(R)*-1** | InChI=1S/C27H18F9N3O2/c28-25(29,30)14-39-20-9-5-4-8-18(20)22(15-6-2-1-3-7-15)38-23(24(39)41)37-21(40)12-16-10-11-17(26(31,32)33)13-19(16)27(34,35)36/h1-11,13,23H,12,14H2,(H,37,40)/t23-/m1/s1 | -51 ± 10 |
|  |  |  |
| **8** | InChI=1S/C25H20F3N3O3/c26-25(27,28)15-31-20-9-5-4-8-19(20)22(17-6-2-1-3-7-17)30-23(24(31)34)29-21(33)14-16-10-12-18(32)13-11-16/h1-13,23,32H,14-15H2,(H,29,33) | -30 ± 3 ^[b]^ |
|  |  |  |
| **10a** | InChI=1S/C25H17F6N3O2/c26-24(27,28)16-11-10-15(18(13-16)25(29,30)31)12-20(35)33-22-23(36)32-19-9-5-4-8-17(19)21(34-22)14-6-2-1-3-7-14/h1-11,13,22H,12H2,(H,32,36)(H,33,35) | +12 ± 2 |
|  |  |  |
| **10b** | InChI=1S/C23H18BrN3O2/c24-18-12-6-4-10-16(18)14-20(28)26-22-23(29)25-19-13-7-5-11-17(19)21(27-22)15-8-2-1-3-9-15/h1-13,22H,14H2,(H,25,29)(H,26,28) | -6 ± 4 |
|  |  |  |
| **10c** | InChI=1S/C22H15BrIN3O2/c23-15-10-14(11-16(24)12-15)21(28)27-20-22(29)25-18-9-5-4-8-17(18)19(26-20)13-6-2-1-3-7-13/h1-12,20H,(H,25,29)(H,27,28) | -14 ± 2 |
|  |  |  |
| **10d** | InChI=1S/C22H18N4O2/c27-19(13-15-7-6-12-23-14-15)25-21-22(28)24-18-11-5-4-10-17(18)20(26-21)16-8-2-1-3-9-16/h1-12,14,21H,13H2,(H,24,28)(H,25,27) | +1 ± 1 |
|  |  |  |
| **10e** | InChI=1S/C22H16N4O4/c27-21(15-9-6-10-16(13-15)26(29)30)25-20-22(28)23-18-12-5-4-11-17(18)19(24-20)14-7-2-1-3-8-14/h1-13,20H,(H,23,28)(H,25,27) | +4 ± 7 |
|  |  |  |
| **12a** | InChI=1S/C32H31F6N3O2/c1-2-3-4-5-11-18-41-26-15-10-9-14-24(26)28(21-12-7-6-8-13-21)40-29(30(41)43)39-27(42)19-22-16-17-23(31(33,34)35)20-25(22)32(36,37)38/h6-10,12-17,20,29H,2-5,11,18-19H2,1H3,(H,39,42) | -4 ± 8 |
|  |  |  |
| **12b** | InChI=1S/C33H31F6N3O2/c34-32(35,36)24-16-15-23(26(20-24)33(37,38)39)19-28(43)40-30-31(44)42(18-17-21-9-3-1-4-10-21)27-14-8-7-13-25(27)29(41-30)22-11-5-2-6-12-22/h2,5-8,11-16,20-21,30H,1,3-4,9-10,17-19H2,(H,40,43) | +1 ± 4 |
|  |  |  |
| **12c** | InChI=1S/C28H22F7N3O2/c29-13-6-14-38-22-10-5-4-9-20(22)24(17-7-2-1-3-8-17)37-25(26(38)40)36-23(39)15-18-11-12-19(27(30,31)32)16-21(18)28(33,34)35/h1-5,7-12,16,25H,6,13-15H2,(H,36,39) | -51 ± 5 |

^[a]^ Activity change of the K_V_7.1 channel in the presence of different concentrations of test compounds referring to buffer only. Activity was recorded by TEVC. Values represent the mean of at least four independent experiments (n ≥ 4).

^[b]^	Compound concentration 10 µM

| **compd.** | **InChICode** |
| --- | --- |
| **3** | InChI=1S/C15H12BrNO2/c16-10-14(18)17-13-9-5-4-8-12(13)15(19)11-6-2-1-3-7-11/h1-9H,10H2,(H,17,18) |
| **4** | InChI=1S/C15H12N2O/c18-14-10-16-15(11-6-2-1-3-7-11)12-8-4-5-9-13(12)17-14/h1-9H,10H2,(H,17,18) |
| **5** | InChI=1S/C17H13F3N2O/c18-17(19,20)11-22-14-9-5-4-8-13(14)16(21-10-15(22)23)12-6-2-1-3-7-12/h1-9H,10-11H2 |
| **6** | InChI=1S/C17H12F3N5O/c18-17(19,20)10-25-13-9-5-4-8-12(13)14(11-6-2-1-3-7-11)22-15(16(25)26)23-24-21/h1-9,15H,10H2 |
| ***rac-*7** | InChI=1S/C17H14F3N3O/c18-17(19,20)10-23-13-9-5-4-8-12(13)14(22-15(21)16(23)24)11-6-2-1-3-7-11/h1-9,15H,10,21H2 |
| ***(R)-*7** | InChI=1S/C17H14F3N3O/c18-17(19,20)10-23-13-9-5-4-8-12(13)14(22-15(21)16(23)24)11-6-2-1-3-7-11/h1-9,15H,10,21H2/t15-/m1/s1 |
| **11a** | InChI=1S/C14H22O3S/c1-3-4-5-6-7-12-17-18(15,16)14-10-8-13(2)9-11-14/h8-11H,3-7,12H2,1-2H3 |
| **11b** | InChI=1S/C15H22O3S/c1-13-7-9-15(10-8-13)19(16,17)18-12-11-14-5-3-2-4-6-14/h7-10,14H,2-6,11-12H2,1H3 |
