# Supplementary material for: Structure–Activity Relationships of 1,4‐Benzodiazepines Inhibiting the Voltage‐Gated Potassium Channel KV7.1
Source: Arch Pharm (Weinheim). 2026 Oct 23;359(10):e70370. doi: 10.1002/ardp.70370 (PMC13691622; doi:10.1002/ardp.70370)
Supplement: Supplementary file 2 [file ARDP-359-e70370-s002.docx]

**Supporting information**

# Structure-activity relationships of 1,4-benzodiazepines inhibiting the

# voltage-gated potassium channel K_V_7.1

Florian Roßner,^(a,b)^ Amy Methaq,^(b)^ Guiscard Seebohm,^(a,c)^ * Bernhard Wünsch^(a,b)^ *

^[a]^	GRK 2515, Chemical biology of ion channels (Chembion), Universität Münster, Corrensstraße 48, D-48149 Münster, Germany

^[b]^	University of Münster, Institute of Pharmaceutical and Medicinal Chemistry, Corrensstraße 48, D-48149 Münster, Germany.

Tel.: +49-251-8333311; E-mail: [wuensch@uni-muenster.de](mailto:wuensch@uni-muenster.de)

^[c]^	University Hospital Münster, Institute for Genetics of Heart Diseases (IfGH), Department of Cardiovascular Medicine, D-48149 Münster, Germany.

Content:	page

1. General methods used in the synthesis part	S2

2. Synthesis of tosylates **11a** and **11b** for alkylation of secondary lactam **10**	S3

3. References	S5

4. NMR spectra	S6

5. HPLC chromatograms showing the purity of the test compounds	S25

**1. General methods used in the synthesis part**

Oxygen and moisture sensitive reactions were carried out under nitrogen, dried with silica gel with moisture indicator (orange gel, VWR, Darmstadt, Germany) and in dry glassware (Schlenk flask or Schlenk tube). Temperature was controlled with ice/water (0 °C), magnetic stirrer MR 3001 K (Heidolph, Schwalbach, Germany) or RCT CL (IKA, Staufen, Germany), together with temperature controller EKT HeiCon (Heidolph) or VT-5 (VWR) and silicone bath. All solvents were of analytical or technical grade quality. Demineralized water was used. CH_2_Cl_2_ was distilled from CaH_2_. Thin layer chromatography (tlc): tlc silica gel 60 F_254_ on aluminum sheets (VWR). Flash chromatography (fc): Silica gel 60, 40–63 µm (VWR); parentheses include: diameter of the column (∅), length of the stationary phase (l) and eluent. Automated flash chromatography: Isolera^TM^ Spektra One (Biotage^®^); parentheses include: cartridge size, eluent, fractions size was always 20 mL. Melting point: Melting point system MP50 (Mettler Toledo, Gießen, Germany), open capillary, uncorrected. MS: MicroTOFQII mass spectrometer (Bruker Daltonics, Bremen, Germany); deviations of the found exact masses from the calculated exact masses were 5 ppm or less; the data were analyzed with DataAnalysis^®^ (Bruker Daltonics). NMR: NMR spectra were recorded in deuterated solvents on Agilent DD2 400 MHz and 600 MHz spectrometers (Agilent, Santa Clara CA, USA); chemical shifts (*δ*) are reported in parts per million (ppm) against the reference substance tetramethylsilane and calculated using the solvent residual peak of the undeuterated solvent; coupling constants are given with 0.5 Hz resolution; assignment of ^1^H and ^13^C NMR signals was supported by 2-D NMR techniques where necessary.IR: FT/IR IR Affinity^®^ spectrometer (Shimadzu, Düsseldorf, Germany) using ATR technique. Optical rotation: Polarimeter 341 (Perkin Elmer); 1.0 dm tube; concentration *c* in g/100 mL; T = 20 °C; wavelength 589 nm (D-line of Na light); the unit of the specific rotation ([α]_D_^T^ grad^.^mL^.^dm^-1.^g^-1^) is omitted for clarity. CD: J600 spectropolarimeter (Jasco) was used for circular dichroism (CD) Spectroscopy. All samples were dissolved in CH_3_OH and the spectra were recorded a 1 mm cuvette. A background correction was performed by subtracting the spectrum of pure CH_3_OH.

**2. Synthesis of tosylates 11a and 11b for alkylation of secondary lactam 10a**

**Heptyl 4-methylbenzenesulfonate (11a) ^[1]^**

At 0 °C, tosyl chloride (923 mg, 4.84 mmol, 1.10 eq.) was added to a mixture of heptan-1-ol (0.62 mL, 511 mg, 4.40 mmol, 1.00 eq.) and pyridine (1.80 mL). The ice bath was removed and the mixture was stirred for 3 h at room temperature. Then, HCl (25 %, 11 mL) was added. The product was extracted with CHCl_3_ (3 x 50 mL). The combined organic layers were dried (Na_2_SO_4_), filtered and concentrated *in vacuo*. Yellow oil, yield 738 mg (2.73 mmol, 62 %). Purity (HPLC): 100.0 % (t_R_ = 25.2 min). Chemical formula: C_14_H_22_O_3_S (270.4 g/mol). TLC: R_f_ = 0.80 (cyclohexane : ethyl acetate 50 : 50). ^1^H NMR (600 MHz, DMSO-d_6_): δ (ppm) = 0.82 (t, *J* = 7.2 Hz, 3H C*H*_3heptyl_), 1.10 – 1.33 (m, 8H, 3-*H*_heptyl_, 4-*H*_heptyl_, 5-*H*_heptyl_, 6-*H*_heptyl_), 1.53 (quint, *J* = 6.7 Hz, 2H, 2-*H*_heptyl_), 2.42 (s, 3H, C*H*_3_-Phe), 4.00 (t, *J* = 6.3 Hz, 2H, 1-*H*_heptyl_), 7.47 (d, *J* = 8.3 Hz, 2H, 3-*H*_Phe_, 5-*H*_Phe_), 7.78 (d, *J* = 8.3 Hz, 2H, 2-*H*_Phe_, 6-*H*_Phe_). ^13^C NMR (151 MHz, DMSO-d_6_): δ (ppm) = 13.8 (C-7_heptyl_), 21.0 (*C*H_3_-Phe), 21.9 (C-6_heptyl_), 24.6 (C-3_heptyl_), 27.9 (C-2_heptyl_), 28.1 (C-4_heptyl_), 31.0 (C-5_heptyl_), 70.9 (C-1_heptyl_), 127.5 (2C, C-2_Phe_, C-6_Phe_), 130.1 (2C, C-3_Phe_, C-5_Phe_), 132.6 (C-1_Phe_), 144.8 (C-4_Phe_). IR (neat): ν̃ (cm^-1^) = 2924 and 2862 (CH_2_), 1597 (C=C, aryl), 1358 and 1173 (O=S=O). Exact mass (APCI): m/z = 271.1347 (calcd. 271.1362 for C_14_H_22_O_3_S [M + H]^+^).

**(2-Cyclohexylethyl) 4-methylbenzenesulfonate (11b) ^[2]^**

At 0 °C, tosyl chloride (923 mg, 4.84 mmol, 1.21 eq.) was added to a mixture of 2-cyclohexylethan-1-ol (0.56 mL, 513 mg, 4.00 mmol, 1.00 eq.) and Et_3_N (1.1 mL, 789 mg, 7.80 mmol, 1.95 eq.) in CH_2_Cl_2_ (16 mL). The ice bath was removed and the mixture was stirred for 1 h at room temperature. Et_2_O (20 mL) was added and the organic layer was washed with 1 M HCl (20 mL) and H_2_O (20 mL). The combined organic layers were dried (Na_2_SO_4_), filtered and concentrated *in vacuo*. The residue was purified by flash chromatography (SiO_2_, cyclohexane : ethyl acetate, gradient 100:0 → 0:100). Colorless oil, yield 576 mg (2.04 mmol, 51 %). Purity (HPLC): 95.9 % (t_R_ = 25.0 min).

Chemical formula: C_15_H_22_O_3_S (282.4 g/mol). TLC: R_f_ = 0.13 (cyclohexane). ^1^H NMR (600 MHz, DMSO-d_6_): δ (ppm) = 0.73 – 0.82 (m, 2H, 2-*H*_ax. cyclohexyl_, 6-*H*_ax. cyclohexyl_), 0.97 – 1.28 (m, 5H, OCH_2_C*H*_2_, 3-*H*_ax. cyclohexyl_, 4-*H*_ax. cyclohexyl_, 5-*H*_ax. cyclohexyl_), 1.39– 1.65 (m, 6H, 1-*H*_cyclohexyl_, 2-*H*_eq. cyclohexyl_, 3-*H*_eq. cyclohexyl,_ 4-*H*_eq. cyclohexyl_, 5-*H*_eq. cyclohexyl_, 6-*H*_eq. cyclohexyl_), 2.42 (s, 3H, C*H*_3_-Phe), 4.03 (t, *J* = 6.4 Hz, 2H, OC*H*_2_CH_2_), 7.47 (d, *J* = 8.2 Hz, 2H, 3-*H*_Phe_, 5-*H*_Phe_), 7.78 (d, *J* = 8.3 Hz, 2H, 2-*H*_Phe_, 6-*H*_Phe_). ^13^C NMR (151 MHz, DMSO-d_6_): δ (ppm) = 21.1 (*C*H_3_-Phe), 25.5 (2C, C-3_cyclohexyl_, C-5_cyclohexyl_), 25.9 (C-4_cyclohexyl_), 32.2 (2C, C-2_cyclohexyl_, C-6_cyclohexyl_), 33.1 (OCH_2_*C*H_2_), 35.4 (C-1_cyclohexyl_), 68.9 (O*C*H_2_CH_2_), 127.6 (2C, C-2_Phe_, C-6_Phe_), 130.1 (2C, C-3_Phe_, C-5_Phe_), 132.4 (C-1_Phe_), 144.9 (C-4_Phe_). IR (neat): ν̃ (cm^-1^) = 2924 and 2847 (CH_2_), 1597 (C=C, aryl), 1358 and 1173 (O=S=O). Exact mass (APCI): m/z = 283.1349 (calcd. 283.1362 for C_15_H_23_O_3_S [M + H]^+^).

**3. References**

[1] Timko, L.; Fischer-Fodor, E.; Garajova, M.; Mrva, M.; Chereches, G.; Ondriska, F.; Bukovský, M.; Lukac, M.; Karlovska, J.; Kubincova, J.; Devínsky, F.; Synthesis of structural analogues of hexadecylphosphocholine and their antineoplastic, antimicrobial and amoebicidal activity, *Eur. J. Med. Chem.,* **2015**, *93*, 263 – 273

[2] Cullen, D.; Gallagher, A.; Duncan, C. L.; Pengon, J.; Rattanajak, R.; Chaplin, J.; Gunosewoyo, H.; Kamchonwongpaisan, S.; Payne, A.; Mocerino, M.; Synthesis and evaluation of tetrahydroisoquinoline derivatives against Trypanosoma brucei rhodesiense, *Eur. J. Med. Chem.,* **2021**, *226*

**4. NMR spectra**

^1^H NMR spectrum of **3** in CDCl_3_

^13^C NMR spectrum of **3** in CDCl_3_

^1^H NMR spectrum of **4** in DMSO-d6

^13^C NMR spectrum of **4** in DMSO-d6

^1^H NMR spectrum of **5** in DMSO-d6

^13^C NMR spectrum of **5** in DMSO-d6

^1^H NMR spectrum of **6** in DMSO-d6

^13^C NMR spectrum of **6** in DMSO-d6

^1^H NMR spectrum of *rac*-**7** in DMSO-d6

^13^C NMR spectrum of *rac-***7** in DMSO-d6

^1^H NMR spectrum of *(R)*-**7** in DMSO-d6

^13^C NMR spectrum of *(R)-***7** in DMSO-d6

^1^H NMR spectrum of **8** in DMSO-d6

^13^C NMR spectrum of **8** in DMSO-d6

^1^H NMR spectrum of *rac-***1** in DMSO-d6

^13^C NMR spectrum of *rac-***1** in DMSO-d6

^1^H NMR spectrum of *(R)-***1** in DMSO-d6

^13^C NMR spectrum of *(R)-***1** in DMSO-d6

^1^H NMR spectrum of **10a** in DMSO-d6

^13^C NMR spectrum of **10a** in DMSO-d6

^1^H NMR spectrum of **10b** in DMSO-d6

^13^C NMR spectrum of **10b** in DMSO-d6

^1^H NMR spectrum of **10c** in DMSO-d6

^13^C NMR spectrum of **10c** in DMSO-d6

^1^H NMR spectrum of **10d** in DMSO-d6

^13^C NMR spectrum of **10d** in DMSO-d6

^1^H NMR spectrum of **10e** in DMSO-d6

^13^C NMR spectrum of **10e** in DMSO-d6

^1^H NMR spectrum of **11a** in DMSO-d6

^13^C NMR spectrum of **11a** in DMSO-d6

^1^H NMR spectrum of **11b** in DMSO-d6

^13^C NMR spectrum of **11b** in DMSO-d6

^1^H NMR spectrum of **12a** in DMSO-d6

^13^C NMR spectrum of **12a** in DMSO-d6

^1^H NMR spectrum of **12b** in DMSO-d6

^13^C NMR spectrum of **12b** in DMSO-d6

^1^H NMR spectrum of **12c** in DMSO-d6

^13^C NMR spectrum of **12c** in DMSO-d6

**5. HPLC chromatograms showing the purity of the test compounds**

HPLC chromatogram of **3** showing the purity of **3** (t_R_ = 20.7 min, method 1).

HPLC chromatogram of **4** showing the purity of **4** (t_R_ = 11.9 min, method 1).

HPLC chromatogram of **5** showing the purity of **5** (t_R_ = 18.5 min, method 1).

HPLC chromatogram of **6** showing the purity of **6** (t_R_ = 23.0 min, method 1).

HPLC chromatogram of *rac-***7** showing the purity of *rac-***7** (t_R_ = 16.6 min, method 1).

HPLC chromatogram of *(R)-***7** showing the purity of *(R)-***7** (t_R_ = 16.5 min, method 1).

HPLC chromatogram of **8** showing the purity of **8** (t_R_ = 19.7 min, method 1).

HPLC chromatogram of *rac-***1** showing the purity of *rac-***1** (t_R_ = 24.9 min, method 1).

HPLC chromatogram of *(R)-***1** showing the purity of *(R)-***1** (t_R_ = 24.9 min, method 1).

HPLC chromatogram of **10a** showing the purity of **10a** (t_R_ = 22.1 min, method 1).

HPLC chromatogram of **10b** showing the purity of **10b** (t_R_ = 19.8 min, method 1).

HPLC chromatogram of **10c** showing the purity of **10c** (t_R_ = 22.8 min, method 1).

HPLC chromatogram of **10d** showing the purity of **10d** (t_R_ = 14.0 min, method 1).

HPLC chromatogram of **10e** showing the purity of **10e** (t_R_ = 19.5 min, method 1).

HPLC chromatogram of **11a** showing the purity of **11a** (t_R_ = 25.2 min, method 1).

HPLC chromatogram of **11b** showing the purity of **11b** (t_R_ = 25.0 min, method 1).

HPLC chromatogram of **12a** showing the purity of **12a** (t_R_ = 27.3 min, method 1).

HPLC chromatogram of **12b** showing the purity of **12b** (t_R_ = 27.2 min, method 1).

HPLC chromatogram of **12c** showing the purity of **12c** (t_R_ = 23.7 min, method 1).
